# Generation and characterization of iPSC‐derived microglia for in vitro modeling of stimuli‐specific neuroimmune responses

**DOI:** 10.1002/alz.71117

**Published:** 2026-02-04

**Authors:** Angela K. Haskell, Joshua A. Kulas, William E. Carter, June Javens‐Wolfe, Raven Dance Hinkel, Mustapha Moussaif, Jacob S. Smiley, Olivia Lazaro, Sylvia Robertson, Alan D. Palkowitz, Bruce T. Lamb, Timothy I. Richardson, Jeffrey L. Dage, Shaoyou Chu, Travis Johnson, Louis F. Stancato, Abdul Qadir Syed

**Affiliations:** ^1^ Indiana Biosciences Research Institute Indianapolis Indiana USA; ^2^ Department of Biostatistics and Health Data Science Indiana University School of Medicine Indianapolis Indiana USA; ^3^ Department of Medicine Division of Clinical Pharmacology Indiana University School of Medicine Indianapolis Indiana USA; ^4^ Department of Medical and Molecular Genetics Indiana University School of Medicine Indianapolis Indiana USA; ^5^ Stark Neurosciences Research Institute IUSM Indianapolis Indiana USA; ^6^ Indiana Alzheimer's Disease Research Center Indianapolis Indiana USA; ^7^ Department of Neurology Indiana University School of Medicine Indianapolis Indiana USA

**Keywords:** immune response, induced pluripotent stem cells, microglia, myelin, phagocytosis, triggering receptor on myeloid cells 2

## Abstract

**INTRODUCTION:**

Microglia are macrophage‐like brain resident immune cells known to express numerous Alzheimer's disease risk genes. Here we generated a human induced pluripotent stem cell (iPSC) derived microglia cell culture model for use in neuroimmune modeling and therapeutic testing.

**METHODS:**

We generated iPSC lines using episomal reprogramming for subsequent stepwise differentiation of iPSC‐derived microglia (iMG) without commercial kits. We characterized the responses of this model to immunogenic stimuli and recombinant TREM2 antibodies.

**RESULTS:**

The iMG expressed several key microglia signature genes and are morphologically and transcriptionally dynamic. iMG rapidly phagocytosed myelin debris and strongly changed expression of lipid homeostasis genes. iMG expressed TREM2 and increased TREM2 levels in response to IL‐4. Recombinant TREM2 antibody treatment impaired iMG myelin phagocytosis and upregulated chemokines.

**DISCUSSION:**

We validated our iMG model system for the evaluation of biological responses of human microglia‐like cells to stimuli and pharmacological agents for their transcriptional and functional impacts.

## Highlights

- Human induced pluripotent stem cell (iPSC) lines were generated from blood cells and differentiated into microglia (iPSC‐derived microglia [iMG]) in a stepwise fashion.
- iMG are morphologically and transcriptionally dynamic in response to diverse immunogenic stimuli.
- Myelin debris treatment induces dramatic changes in microglial lipid metabolism and induces the formation of lipid droplets.
- IL‐4 increases the expression of TREM2 and DAP12 in iMG but does not enhance myelin uptake.
- TREM2 agonist antibody reduces myelin phagocytosis while upregulating CCL1 and CCL22.

## 1 BACKGROUND

Microglia are highly specialized brain resident immune cells and professional phagocytes which originate from primitive macrophages that enter circulation and migrate into the brain during development.^1^, ^2^ Several lines of evidence suggest microglia play an important role in the pathogenesis of Alzheimer's disease (AD). Established AD risk genes, such as TREM2, PLCG2, INPP5D, and CD33, are highly expressed in microglia and regulate microglial functional responses to amyloid plaques in transgenic animal models.^3^, ^4^ Disruption of microglial signaling genes, including SYK, CARD9, INPP5D, and PLCG2, impacts brain amyloid plaque burden and microglial responses to amyloid deposits.^5^, ^6^, ^7^, ^8^ Interestingly, chemical depletion of microglia from transgenic animal models is sufficient to prevent brain parenchymal amyloid plaque accumulation, suggesting microglia are required for the production or initial generation of brain amyloid deposits.^9^ Taken together, these data are suggestive of the therapeutic potential of microglia targeted approaches as treatments for AD; therefore, harnessing the potential neuroprotective functions of microglial cells remains compelling as a therapeutic strategy.^10^ Despite this, practical challenges remain in developing reproducible model systems that accurately mimic human microglia for the characterization and optimization of pharmacological agents and therapeutic development. For example, fundamental differences exist between mouse and human microglia, and microglia cell lines derived from mice have limited translational potential.^11^, ^12^

As part of the TREAT‐AD Center's mission to provide high‐quality research tools and technologies to validate and advance the next generation of AD drug targets, we describe the generation of human iPSC lines from erythroid progenitor cells cultured from blood peripheral blood mononuclear cells (PBMC) fractions using non‐integrating episomal reprogramming factors. These iPSC lines are subsequently differentiated into microglia (iMG) through a well‐defined, stepwise differentiation method designed to mimic primitive hematopoiesis using small molecules and human recombinant proteins to target specific microglial developmental pathways. We rigorously validate the iMG generated using this protocol and perform several experiments to induce the cells into specific activation states. We characterize the iMG response to myelin debris and find that myelin debris drives significant transcriptional changes in cellular lipid metabolism genes to control cellular lipid levels, including the formation of PLIN2 lipid droplets. We find that iMG robustly express TREM2 and that expression of both TREM2 and DAP12 can be increased by interleukin (IL) ‐4 treatment, though this does not enhance myelin phagocytosis, suggesting TREM2 levels are not a limiting factor in myelin uptake.

**RESEARCH IN CONTEXT**

1. **Systematic review**: The authors performed a comprehensive review of existing literature on induced pluripotent stem cell (iPSC) microglia models using PubMed as the primary search platform. Several studies generate iPSC derived microglia (iMG) using commercial kits and do not rigorously assess the functional characteristics of these cells. Here, we provide a multi‐assay characterization of iPSC microglia differentiated with defined factors from a newly generated iPSC line and utilize the cells to study a TREM2 agonist antibody.
2. **Interpretation**: Our stepwise differentiation protocol consistently generates iMG with properties similar to human microglia including ramified morphology, rapid phagocytosis and expression of microglial signature genes including IBA1, TMEM119, HEXB, and TREM2. These iMG exhibit discrete responses to relevant stimuli and significantly shift lipid metabolism when challenged with myelin debris. iMG induce consistent transcriptional signatures when treated with a TREM2 agonist antibody revealing a potential role for TREM2 in inducing chemokine expression.
3. **Future directions**: Our data suggest that iMG have functional similarities to human microglia, and future studies will incorporate this model system to better understand the potential of therapeutic agents targeting microglia and the neuroimmune system.

Reliable human microglia models may be of particular value in understanding the transcriptional responses of microglia to molecules, as large differences exist in the transcriptional profiles of microglia across species.^13^ To evaluate the potential of iMG as a platform to test emerging therapeutics, we generated a recombinant TREM2 agonist antibody and used this model system to evaluate its impact on microglial transcriptional state and myelin phagocytosis. Surprisingly, the antibody bound iMG cells and induced a specific chemokine response while significantly reducing acute myelin debris uptake. We conclude that iMG generated using the method described here are a useful model to explore how pharmacological agents targeting microglia may impact microglial transcriptional states and functional characteristics. This model system will be used to inform and supplement testing strategies and obtain new insights into the potential biological effects of these molecules on human microglia.

## 2 METHODS

### 2.1 Generation of human iPSC lines

Erythroid progenitor cells were isolated and reprogrammed from human peripheral blood mononuclear cells (PBMCs) obtained from BioIVT using the STEMCELL Technologies Erythroid Progenitor Reprogramming Kit (STEMCELL Technologies, 05924) and Epi5 Episomal iPSC Reprogramming Kit (Invitrogen, A15960). Both iPSC lines described in this study were derived from a healthy female donor. A 10 mL blood sample was equilibrated to room temperature in a 50 mL conical vial. 50 µL of RosetteSep Human Progenitor Cell Basic Pre‐Enrichment Cocktail was added directly to the undiluted blood, mixed gently by serologic pipet, and incubated at room temperature for 10 min. The blood sample was diluted 1:1 with PBS + 2% FBS. 15 mL of Lymphoprep was added to the SepMate‐50 mL tube through the central hole of the insert. The diluted blood sample was then added to a SepMate tube containing Lymphoprep gently down the wall of the tube to layer the blood sample on the top. The sample was centrifuged for 20 min at 1200 × g. Enriched cells were collected by pouring the top layer quickly into a new tube. These cells were washed by adding enough phosphate buffered saline (PBS) + 2% fetal bovine serum (FBS) to fill the tube and centrifuging at 300 x g for 8 min. The cells were resuspended in erythroid expansion medium with supplement and plated at 5 × 10^5^ cells in 2 mL per well (STEMCELL Technologies, 09605, 02692). After 24 h, the 2 mL cell suspension was transferred to a new well, and on days 2, 4, and 6, the cells were collected, centrifuged at 300 x g, resuspended and reseeded in 2 mL of Erythroid Expansion Medium per well of a six‐well plate. At day 7, suspended cells were centrifuged and resuspended in StemPro‐34 serum‐free medium (SFM) containing 100 ng/mL stem cell factor (SCF), 50 ng/mL IL‐3, and 25 ng/mL macrophage colony‐stimulating factor (M‐CSF) (Table S1). Three days later, cells were combined with the Epi5 reprogramming vectors and electroporated using the Lonza 4D‐Nucleofector system. After coating a six‐well plate with growth factor reduced Matrigel basement membrane matrix (Corning, 35‐6231) for 2 h at room temperature, the coating was aspirated and cells were seeded in StemPro‐34 SFM containing cytokines for 24 h. Cells were fed every day by carefully removing 1 mL of media without disturbing the cells and adding 1 mL of N2B27 Medium (Table S2). After 9 days, the media was aspirated and replaced with Essential 8 Medium (Gibco, A1517001). 15 to 21 days after electroporation, iPSC colonies appeared and were manually selected. IBRI 104.G and IBRI 104.B iPSC lines were karyotyped by WiCell at passage number 13 and 9, respectively.

### 2.2 Maintenance of iPSCs

iPSC lines were grown on six‐well plates coated with growth factor reduced Matrigel basement membrane matrix and cultured in Essential 8 Medium (Gibco, A1517001). Colonies were grown to 65%–70% confluency before being passaged with 0.5 mM ethylenediaminetetraacetic acid (EDTA; Invitrogen, 15575‐038) diluted in Dulbecco's PBS (DPBS; Gibco, 14190‐144). Cells were passaged in Essential 8 Medium with 10 µM rho‐associated kinase (RHO/ROCK) pathway inhibitor Y‐27632 (dihydrochloride) (Stemcell Technologies, 72304) with a 1:6 split ratio. Cells were refreshed with Essential 8 Medium (without inhibitor) 24 h after passaging, and daily after that. iPSC colonies were typically passaged twice weekly. iPSCs were tested for mycoplasma using a commercially available kit (InvivoGen, rep‐pt1).

### 2.3 Embryoid body formation

To form embryoid bodies, iPSCs were cultured to 60%–70% confluency and disassociated using 0.5 mM EDTA. Cells were resuspended in Essential 8 Medium with 10 µM Y‐27632 ROCK inhibitor and seeded in a low‐attachment six‐well plate (Corning, 3471). iPSCs were gently placed on a shaking platform (100 rpm) in a cell culture incubator for 24 h. Aggregates appeared overnight and the media was changed every other day by centrifuging the aggregates at 300 × g for 3 min and resuspending them in 4 mL of hES media (Table S3). After 7 days, embryoid bodies were collected and seeded in a Matrigel‐coated eight‐chamber immunohistochemistry slides in hES media where they were allowed to adhere and spontaneously differentiate. hES media was refreshed every other day and cells were fixed after 7 days to identify the formation of the primary germ layers by immunofluorescence.

### 2.4 Derivation of hematopoietic progenitor cells from human iPSCs

Throughout the differentiation process, basal media was warmed to 37°C in a bead bath and each growth factor was added fresh just before feeding the cells. Factors were aliquoted in single‐use volumes to avoid multiple freeze–thaw cycles. Media components and added factors are listed in supplemental tables (Tables S4–S6). The method for differentiating iPSCs to hematopoietic progenitor cells (iHPCs) was adopted from a previously published protocol.^14^ Briefly, iPSCs were cultured on Matrigel coated plates until they reached a confluence of 70%. Cells were then passaged with 0.5 mM EDTA and resuspended in 1.4 mL of Essential 8 Medium with 10 µM ROCK inhibitor. 20 µL of the cell suspension were seeded into each well of a Matrigel coated six‐well plate into 2 mL of Essential 8 Medium with 10 µM Y‐27632 ROCK inhibitor. After 24 h, the seeding of iPSCs yielded 20–40 colonies per well with a diameter of 150–250 µm. If the colonies were too small (< 100 µm), the media was refreshed with 2 mL of Essential 8 Medium (without ROCK inhibitor) and cells were incubated overnight to allow colony expansion. Any colonies above 500 µm were scraped from the well. Once the colonies were the appropriate size and number (day 0), the media was refreshed with 2 mL per well of iHPC media containing 25 ng/mL BMP‐4, 15 ng/mL Activin A, and 1.5 µM CHIR99021. On day 2, each well is refreshed with 3 mL per well of iHPC media containing 50 ng/mL vascular endothelial growth factor (VEGF), 50 ng/mL basic fibroblast growth factor (bFGF), 10 µM SB431542, and 50 ng/mL SCF. On day 5 and day 7, each well is refreshed with 2 mL of iHPC media containing 50 ng/mL VEGF, 50 ng/mL bFGF, 50 ng/mL SCF, 10 ng/mL IL‐3, 50 ng/mL IL‐6, and 50 ng/mL thrombopoietic (TPO) (Table S6). After 5 to 7 days, small bright non‐adherent cells begin to emerge and lift from the adherent colonies. At day 9, the non‐adherent cells suspended in the media were gently collected and taken for characterization of surface antigens and moved to the next stage of the differentiation.

### 2.5 Differentiation of iHPCs to iMG

The method used to differentiate iPSC‐derived iHPCs to iMG was adopted from a previously published protocol.^15^ Briefly, on day 9 of the differentiation process, iHPCs were harvested from the media by washing the media over the well. This was done gently to avoid the detachment of any adherent cells, which led to off‐type cells in the next stage of the differentiation. Once collected, iHPCs were centrifuged at 300 × g for 5 min and resuspended at 250,000 cells/mL of iMG media containing 100 ng/mL IL‐34, 50 ng/mL transforming growth factor beta (TGF‐β), and 25 ng/mL M‐CSF (Tables S5–S6). These were seeded onto fresh Matrigel coated six‐well plates at 2 mL of cell suspension per well (approximately 50,000 cells/cm^2^). 48 h later, on day 11 of the differentiation, 1 mL of iMG media containing IL‐34, TGF‐β, and M‐CSF was added to each well. On day 13 and every 48 h thereafter, 1 mL of expended media was gradually removed from each well by pipetting slowly from the periphery of the well, drawing from the very surface of the media to not disturb the settled cell population. 1 mL of fresh iMG containing cytokines was added to each well. On day 33 of the differentiation, all media was collected from each well and 1 mL of iMG media containing 100 ng/mL IL‐34, 50 ng/mL TGF‐β, 25 ng/mL M‐CSF, 100 ng/mL CX3CL1, and 100 ng/mL CD200 was added to each well. The collected media was centrifuged at 300 × g for 5 min to rescue any non‐adherent cells, resuspended in 1 mL per well of iMG media containing IL‐34, TGF‐β, M‐CSF, CX3CL1, and CD200, and added back into each well of iMG. On day 35 of the differentiation, 1 mL of iMG media containing IL‐34, TGF‐β, M‐CSF, CX3CL1, and CD200 was added to each well. After day 36 of the differentiation, iMG were used for assays, or kept in culture up to 2 weeks by gently removing and adding 1 mL of iMG media containing IL‐34, TGF‐β, M‐CSF, CX3CL1, and CD200 (Table S6).

### 2.6 Myelin debris and *Staphylococcus aureus* phagocytosis assays

iMG were released from the Matrigel coated six‐well plates on which they were differentiated by accutase treatment (Fisher Scientific, A1110501), quenching with basal media. Resuspended iMG cells were then seeded directly on PhenoPlate 96‐well microplates (Revvity, 6055302) for at least 24 h prior to the initiation of the phagocytosis assay. pHrodo labeled myelin debris was generated using previously described methods.^16^ All phagocytosis assays were conducted in phenol‐red free iMG media containing IL‐34, TGF‐β, M‐CSF, CX3CL1, and CD200. 1 mg/mL phrodomyelin debris was thawed and wand sonicated, then applied to iMG at a final protein concentration of 5 µg/mL. *S. aureus* pHrodo bioparticles (ThermoFisher Scientific, A10010) were applied to iMG at concentration 20 µg/mL. Cytochalasin D (MilliporeSigma, C2618) was utilized at a concentration of 5 µM to inhibit phagocytosis. Upon treatment, imaging plates were rapidly transferred to an Operetta CLS high content imaging system (Revvity) set to 37°C and 5% CO_2_. iMG were imaged every 30 min in brightfield, digital phase contrast, and rhodamine channels using a 20x objective for 4 h to measure changes in cellular fluorescence. Analysis of phagocytosis was conducted using Harmony software v4.9 (Revvity) to segment individual iMG cells and quantify the mean pHrodo fluorescence within the iMG cellular area as defined using the digital phase contrast signal. The mean cellular fluorescence of the microglia population at each timepoint within a well was calculated as an individual data point in each respective experiment. All phagocytosis assays were repeated with at least 3 independent differentiation cultures of iMG and representative experiments are shown. When testing the effect of IL‐4 on phagocytosis, IL‐4 (Peprotech, 200‐04) was applied to iMG at a concentration of 50 ng/mL for 24 h before initiation of the phagocytosis assay. When testing the effects of the TREM2 antibody and the control IgG antibody, iMG were pretreated with antibodies at a concentration of 1 ug/mL for 30 min before the initiation of the phagocytosis assay.

### 2.7 High content imaging—microglia morphology analysis and % positive cells

Fixed iMGs stained with Hoechst and CellMask Green (Invitrogen) were imaged using the Operetta CLS high content imaging system (Revvity) with a 10x air objective (NA = 0.3) and 2 × 2 pixel binning. Images were analyzed using Harmony v4.9 software. Nuclei were segmented based on the gaussian smoothed Hoechst channel using the Find Nuclei block. Cell boundaries for each nucleus were determined using the Find Cytoplasm block on the CellMask channel. Cell types were defined using the Select Population block analyzing only cells that were not touching the edges of the image. Elongated cells were defined as having a cell width of < 23 µm and a roundness of < 0.75 µm. Ameboid cells were defined as having a cell width of > 30 µm, a cell roundness of < 0.865 µm, and a cell length‐to‐width ratio of > 0.35. Note that Harmony defines cell width as the diameter of the largest circle that can fit within a shape. The percentage of round or ameboid cells out of the total cell count from nine fields of view was calculated and graphed as an average of seven replicated wells per treatment.

For quantification of immunofluorescent cells, iMGs labeled with anti‐P2RY12R (Abcam, Clone 1C2A9), anti‐PU.1 (Cell Signaling, 89136S), or recombinantly produced anti‐TREM2 antibodies were imaged using the Operetta CLS high content imaging system (Revvity) with a 20x air objective and analyzed using the corresponding Harmony v4.9 software. Cells were segmented from digital phase contrast images using the “Find Surrounding Region” analysis block linked to the nuclei population previously segmented using 4′,6‐diamidino‐2‐phenylindole (DAPI) staining. For P2RY12R and TREM2 labeling, the “Calculate Intensity Properties” block was used in the appropriate secondary antibody channel within the previously defined surrounding region for each nuclei. For PU.1 labeling, the “Calculate Intensity Properties” block was used within the nuclei population region as no staining was present outside the nucleus. Representative images from wells labeled only with secondary antibody (P2RY12R and PU.1) or with an isotype control IgG (TREM2 antibody) were used to define the threshold between negative‐ and positive‐staining cells.

### 2.8 Reverse transcription quantitative polymerase chain reaction

RNA for quantitative polymerase chain reaction (qPCR) experiments were collected using a Quick‐RNA Miniprep Kit (Zymo Research, R1054 R1055). RNA concentrations were determined in water by nanodrop absorbance and normalized across all samples in each experiment before cDNA synthesis. Reverse transcription was performed by Maxima First Strand cDNA synthesis kit (Thermo Scientific, K1642). RT‐qPCR was performed using SYBR green reagent in 384‐well plates using a QuantStudio 5 instrument. Target mRNA expression was determined using the ΔΔCT formula with RPL13 used as a reference gene in all qPCR experiments. Undifferentiated iPSC lines were used as negative control RNA for microglial targets. A list of all primer sequences used in this study is included as a supplemental material (Tables S7).

### 2.9 Western blot

iMG were lysed in radioimmunoprecipitation assay (RIPA) buffer (MilliporeSigma, R0278) in the presence of protease and phosphatase inhibitor (ThermoFisher Scientific, 78429). Protein content was determined by bicinchoninic acid (BCA) assay (Pierce, 23227). 5 micrograms of iMG cell lysate protein were resolved using Stain‐Free 4%–15% acrylamide gels (Biorad, 4568086) and total protein levels in gels were imaged before transferring to a PVDF membrane (Invitrogen, IB24002) for subsequent western blots. Polyvinylidene difluoride (PVDF) membranes were blocked with milk protein (RPI, M17200) diluted in PBS containing 0.1% Tween‐20 detergent (Promega) before application of antibodies. DAP12 antibody (Cell Signaling, E7U7T) was used at a 1:500 dilution in 5% milk. PLIN2 antibody (MilliporeSigma, SAB4200452) was used at a 1:500 dilution in 5% milk solution. Actin‐rhodamine antibody (Biorad, 12004164) was used as a loading control at a 1:5000 dilution in milk solution. Densitometry analysis of western blot protein was performed by calculating the integrated density of bands of interest using Adobe Photoshop software and normalized to their respective actin band intensity as a loading control.

### 2.10 Immunofluorescence

For immunofluorescence, iPSC, three germ layers (EBs), and iMG were fixed by incubation with 4% paraformaldehyde for 20 min at room temperature. Fixed cells were washed with PBS, then blocked and permeabilized with a PBS blocking solution containing 1% Triton X‐100, 5% BSA, and 5% fetal bovine serum. Pluripotency was assessed by immunostaining for TRA‐1‐60, NANOG, SSEA‐4, and OCT4 using the Abcam kit (Abcam, ab109884) according to the manufacturer's instructions. Differentiation into the three primary germ layers was confirmed by staining for ectodermal, endodermal, and mesodermal markers: β‐III‐Tubulin (Promega, G712A), SOX17 (R&D Systems, AF1924), and Vimentin (Cell Signaling Technology, #5741S), respectively. Recombinant TREM2 and IgG antibodies were used at a 2 µg/mL concentration for immunostaining. IBA1 antibody was used at 1:500 (Fujifilm, 019‐19741) dilution in PBS solution described above. Fluorescent anti‐human IgG Alexa Fluor 647 (Invitrogen, A56019), anti‐rabbit immunoglobulin G (IgG) Alexa Fluor 647 (Invitrogen, A32795), anti‐rabbit IgG Alexa Fluor 488 (Abcam, ab150073), anti‐mouse IgG Alexa Fluor 647 (Abcam, ab150117), and anti‐goat IgG Alexa Fluor 594 (Abcam, ab150136) secondary antibodies were diluted in PBS blocking solution at a 1:1000 or 1:250 dilution. DAPI counterstain in PBS was used to visualize cell nuclei.

### 2.11 Lipid droplet imaging

iMG cultures were seeded in 96‐well imaging plates and stimulated with myelin debris for 24 h and then fixed with 4% paraformaldehyde in PBS for 20 min. Lipidspot 610 (Biotum, 70069) dye was applied to iMG in PBS solution at a 1:1000 dilution and then washed 3 times with PBS before imaging. Mean cellular fluorescence was imaged and quantified using the Operetta CLS high content imaging system with Harmony software v4.9 (Revvity).

### 2.12 Generation of recombinant TREM2 agonist antibody

The agonist anti TREM2 mab AL2p‐31 heavy and light chain variable DNA sequences^17^ were synthetized as gene fragments and subcloned separately into digested vectors using In‐Fusion HD cloning enzyme mix (Takara Bio). The vector expressing the wild‐type (WT) human IgG1 heavy chain was mutated with the Quikchange Lightning multi‐site directed mutagenesis kit (Agilent) to generate the LALAPG Fc variant, (includes three‐point mutations L234A, L235A, and P329G) known to abrogate binding to FcγRs and C1q while FcRn binding and Fc stability remain unaffected.^18^ Following vector sequence confirmation, the verified heavy chain and light chain plasmids were co‐transfected at equal ratios and transiently expressed in the freestyle Chinese hamster ovary cells adapted for growth in suspension cultures (CHO‐S) cells using the ExpiFectamine CHO Transfection Kit (Gibco A29129) according to the manufacturer protocol. Transfected cells were grown for 11–14 days and harvested by centrifugation at 8000 rpm for 30 min to recover the supernatant fraction, which was then passed through a 0.22 µm filter. The IgG1 titer was determined by BLI analysis (Octet) using protein G biosensors (Sartorius) and the human IgG1‐Kappa‐UNLB (SouthernBiotech cat number 0151K‐01). The human LALAPG mutant IgG1 was then purified from the filtered supernatants by affinity chromatography by loading onto HiTrap MabSelect PrismA columns packed with high‐capacity Protein A agarose resin (Cytiva 17549853). Unbound proteins were washed away with at least 10x column volume of PBS. All antibodies were eluted with 100 mM citrate buffer (pH 3.0) and immediately neutralized with 1 M Tris (pH 8.0). Samples were then desalted using Zeba Spin Desalting Columns (Thermo Scientific, 89882). Antibodies were loaded in lithium dodecyl sulfate (LDS) sample buffer (Thermo Fisher Scientific, NP0008) with or without 1x sample reducing agent (Thermofisher, 77720) and purity was assessed to be equal or higher than 90% by sodium dodecyl sulfate‐polyacrylamide gel electrophoresis (SDS‐PAGE) with precast NuPAGE 4 to 12%, Bis‐Tris, Mini Protein Gels (Invitrogen, cat number NP0323BOX). An Fc mutant isotype IgG1 antibody with non‐relevant heavy and light chain variable sequences was made similarly and used as negative control.

### 2.13 Flow cytometry

iHPCs were collected and resuspended in DPBS (without calcium or magnesium) on ice with a cell density of 200,000 cells per tube. A control tube was incubated in the presence of 50% ethanol for 10 min. Sample tubes were then stained with Live/Dead fixable violet dead kit (ThermoFisher, L34955) for 30 min on ice. All tubes were then centrifuged at 300 × g for 5 min at 4°C. Cells were resuspended in eBioscience flow cytometry staining buffer (Invitrogen, 00‐4222‐26) and stained with 1 µg/mL of CD34‐FITC (Biolegend, 343604) and CD43‐APC (Biolegend, 343206). UltraComp eBeads Plus Compensation Beads (Invitrogen, 01‐333‐342) were used as a single‐color compensation control for both CD34‐FITC and CD43‐APC, and ArC amine reactive compensation beads (Invitrogen, A10628) were used for the Live/Dead. Fluorescence Minus One (FMO) controls were used for the gating strategy.

### 2.14 Lipopolysaccharide, interferon‐γ, IL‐4 immunostimulant assay

iMG were released by Accutase treatment, resuspended in iMG media containing IL‐34, TGF‐β, M‐CSF, CX3CL1, and CD200, and seeded directly on a tissue culture‐treated 24‐well plate (Corning, 3524) at least 24 h before the stimulation of iMG. iMG were treated with 20 ng/mL of lipopolysaccharide (LPS) (Sigma‐Aldrich, L4391), 100 ng/mL interferon‐γ (IFN‐γ) (Peprotech, 300‐02), both LPS and IFN‐γ, or 50 ng/mL of IL‐4 (Peprotech, 200‐04), with untreated iMG as a control. After 24 h of stimulation, media was collected to analyze cytokine secretion and iMG were lysed for RNA to perform RT‐qPCR. This was performed in iMG from both IBRI 104.G and IBRI 104.B iPSC lines. Measurements of cytokines in conditioned media was conducted using ProQuantum PCR based proximity ligation assay kit (ThermoFisher, A35578) following manufacturer instructions. Media was diluted 1:5 for analysis.

### 2.15 Total RNA sequencing and data analysis

Four independently cultured iMG batches were plated in 96‐well imaging plates. iMG were stimulated with either the TREM2 agonist antibody (1 µg/mL), IgG antibody (1 µg/mL), myelin debris (5 µg/mL), TREM2 agonist antibody with myelin debris, or IgG with myelin debris, using untreated iMG as a control. The cells were imaged using high‐content imaging at time 0 and after 24 h of incubation to ensure the health and quality of the cells. iMG were subsequently lysed and RNA was isolated using the Zymo Research Quick‐RNA miniprep kit (Zymo Research, R1054, R1055).

Analysis was performed in RStudio version 4.3.1. The R package DESeq2 version 3.21 was used for differential gene expression analysis using the counts generated during alignment and analyses followed the standard DESeq2 workflow^19^ using the treatment for each group as conditions for experimental design. Differentially expressed genes were designated as significant with a log2FC > 0.58 or < −0.58 and with a *p*‐value < 0.05. Volcano plots were generated with the R package ggplot2^20^ and heatmaps were generated using the R package heatmap3.^21^ Pathway analysis was performed using the standard clusterProfiler workflow^22^ and used the ReactomePA package^23^ for pathway enrichment analysis. Raw and processed data have been uploaded to GEO (GEO Accession Number GSE306820).

### 2.16 Statistical analysis

Statistical analysis was performed using GraphPad Prism V10. Statistical significance is denoted in figures as *p < 0.05; **p < 0.01; ***p < 0.001 and ****p < 0.001. Tukey's post host test was used for multiple comparisons when significance was identified by one‐way analysis of variance (ANOVA).

### 2.17 Cell line availability

The IBRI 104.G and IBRI 104.B iPSC lines are maintained by the Indiana Biosciences Research Institute and are available for research use upon request. Requests for the 104.G and IBRI 104.B iPSC line can be directed to the IBRI iPSC laboratory by email at: asyed@indianabiosciences.org

## 3 RESULTS

### 3.1 Generation of iPSC lines

**FIGURE 1:**
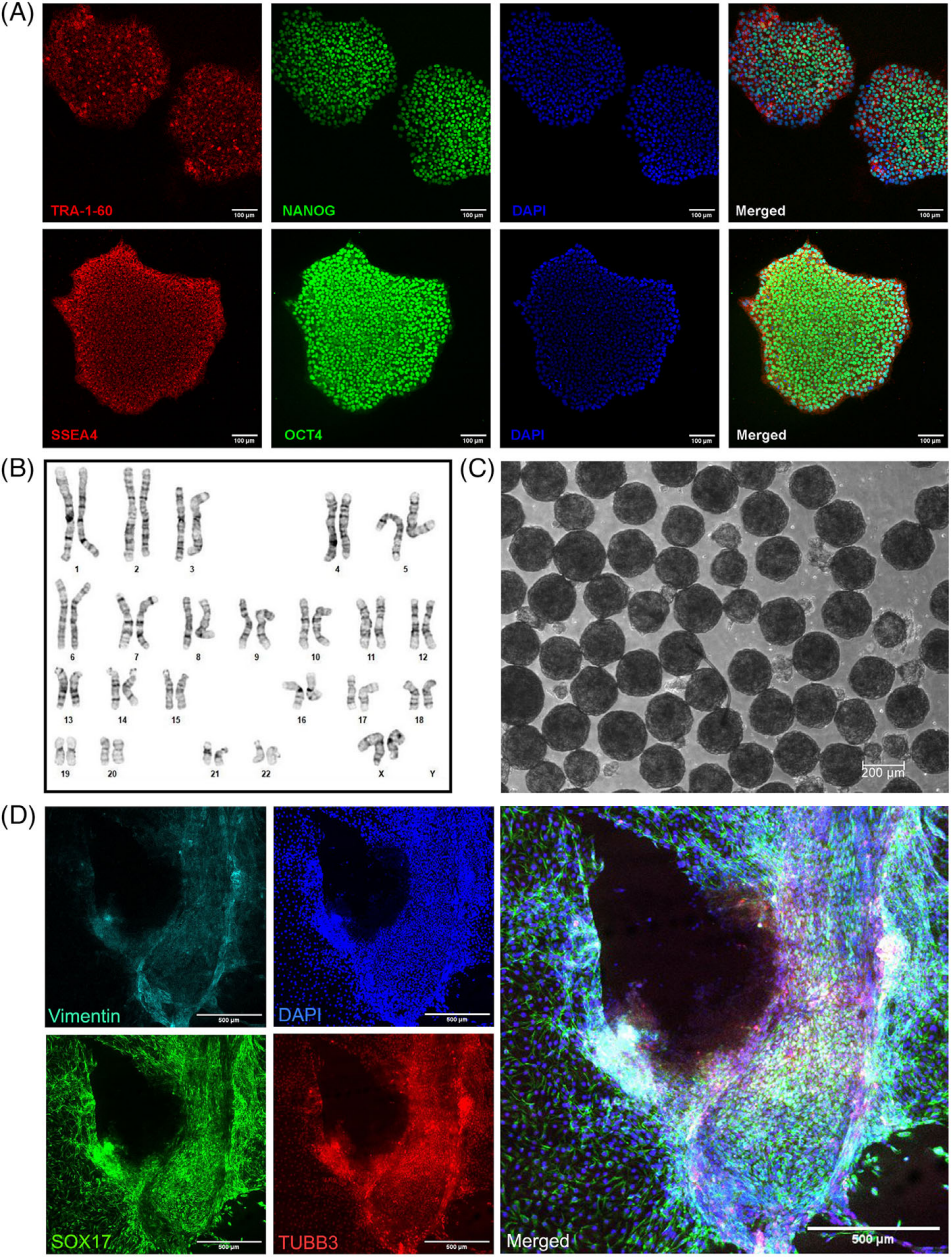
Characterization of the IBRI 104.G iPSC line. (A) Fluorescent images of IBRI 104.G iPSC colonies stained with antibodies against TRA‐1‐60, NANOG, SSEA4 and OCT4 with a DAPI DNA counterstain. Scale bar, 100 µm. (B) Karyotyping of the IBRI 104.G iPSC line. (C) Brightfield images of embryoid bodies generated by the IBRI 104.G line. Scale bar, 200 µm. (D) IBRI 104.G iPSCs spontaneously differentiated into the primary germ layers stained with antibodies against Vimentin (mesoderm), SOX17 (endoderm), and β‐III‐Tubulin (ectoderm). Scale bar, 500 µm.

We utilized the Epi5 episomal reprogramming kit to generate iPSC lines from human erythroid progenitor cells cultured from blood PBMC somatic cells. Individual colonies were selected for further validation, and selection of two clonal colonies resulted in the generation of the IBRI 104.G and the IBRI 104.B iPSC lines. The colonies displayed typical iPSC characteristics, including tightly packed colonies with refractile borders and cells with a high nuclear‐to‐cytoplasmic ratio. These cells were analyzed using immunofluorescence for several stem cell markers, including surface antigens SSEA4 and TRA‐1‐60, in addition to transcription factors NANOG and OCT4 (Figure 1A). Pluripotent stem cells are known to possess the potential to differentiate into any of the three germ layers. To further validate the pluripotent status of our iPSC lines, we performed embryoid body (EB) analysis and observed the size and shape of EBs formed using brightfield microscopy (Figure 1C) and immunofluorescence to identify the proteins vimentin (mesoderm), β‐III‐tubulin (ectoderm), and the transcription factor SOX17 (endoderm) (Figure 1D). The cell line IBRI 104.G has a normal karyotype at passage 13 (Figure 1B) with two X chromosomes confirming the cell line is female. Identification of pluripotency markers and EB analysis were also performed on cell line IBRI 104.B, which revealed a normal female karyotype (Figure S1).

### 3.2 Differentiation of iPSCs to iMG

**FIGURE 2:**
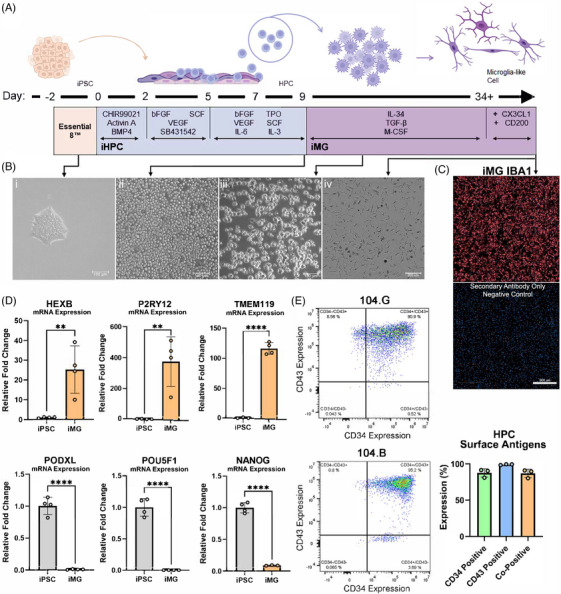
Generation of iMG from iPSCs. (A) Schematic illustrating 34+ days of iMG differentiation from iPSCs created with BioRender.com. (B) Brightfield images of the stages of iMG differentiation: i. iPSC colony on day 0, ii. Hematopoietic progenitor cells on day 9 of the differentiation, iii. Differentiating iMG after 8 days in microglia induction media (day 17 of the differentiation), scale bar 100 µm, iv. iMG on day 36 of the differentiation, scale bar 200 um. (C) Fluorescent images of IBRI 104.G iMG stained with antibodies against IBA1. Scale bar, 500 um. (D) Relative mRNA expression of microglial genes and iPSC genes measured by qPCR. Data are represented as mean ± SD. **p ≤ 0.01; ****p ≤ 0.0001 by unpaired t test. (E) Flow cytometry plots of HPCs generated from both IBRI 104.G (upper panel) and IBRI 104.B (lower panel) iPSCs. The percentage of cells expressing HPC surface antigens in three independent differentiations is graphed.

Our goal was to develop and validate a reproducible method to generate iMG from iPSC lines without the need for commercially developed kits, hypoxic cell culture conditions, or cell sorting. We based our differentiation protocol on previously developed methods which identified key differentiation factors required to mimic microglia ontogeny by first undergoing primitive hematopoiesis, then further development into microglia, directed by the addition of growth factors and selective inhibition of discrete signaling pathways.^11^, ^14^, ^15^, ^24^, ^25^, ^26^ Our protocol (Figure 2A) mimics the differentiation of primitive HPCs followed by the induction of primitive microglia and then a homeostatic population of microglia‐like cells in astrocytic proteins. Using brightfield microscopy, we monitored the morphology changes in the differentiation over time beginning at iPSC colonies through the differentiation of ramified microglia‐like cells (Figure 2B). iPSC derived microglia immunolabeled strongly for the common microglia neuropathology marker IBA1 (Figure 2C). To further validate the microglial identity of these cells, we measured three established human microglial genes, *HEXB, P2RY12*, and *TMEM119* using the parent iPSC line RNA as a control. Furthermore, iMG differentiation significantly reduced expression of pluripotency genes NANOG, PODXL, and POU5F1 (Figure 2D). Flow cytometry characterization confirmed the efficiency of this method in generating CD34^+^/CD43^+^ cells from both iPSC lines (Figure 2E). To further validate the microglial identity of the differentiated cell populations, we conducted immunofluorescent labeling and high‐content imaging of the differentiated iMG labeled for microglial proteins P2RY12, PU.1 and TREM2. (Figure S2A‐S2D) All three microglial proteins were found to be present in greater than 98% of differentiated iMG cultures. Together, these data support the successful generation of a well‐defined, stepwise differentiation of iMG from newly established iPSC lines.

### 3.3 Functional validation of iMG cytokine responses

Microglia are known for their ability to adopt a variety of context specific transcriptional states in response to environmental stimuli such as infectious antigens or molecular signals, including cytokines and chemokines.^27^ To evaluate the transcriptional plasticity of our iMG cells, we stimulated iMG generated from both IBRI 104.G and IBRI 104.B iPSC cell lines with established inflammatory stimuli including bacterially derived LPS (20 ng/mL), IFN‐γ cytokine (100 ng/mL), or LPS & IFN‐γ combined. We also stimulated iMG with IL‐4, a multifunctional cytokine noted for its role in tissue repair among a variety of other biological functions.^28^ After 24 h of stimulation, we measured the transcription of cytokines known to be expressed under inflammatory conditions including *TNFα* and *IL1β* (Figure S3A, S3B). *TNFα* mRNA levels were significantly increased in both cell lines in response to IFN‐γ, and the combination of LPS and IFN‐γ induced a greater upregulation of *TNFα* than either IFN‐γ or LPS alone. Similarly, iMG *IL1β* mRNA levels were significantly upregulated in both iMG cell lines in response to IFN‐γ and further upregulated following combined LPS and IFN‐γ stimulation. CXCL10 is known to be induced by IFN‐γ and its mRNA levels were measured to further test the IFN‐γ cytokine responsiveness of the iMG cell lines. iMG differentiated from both iPSC lines demonstrated a robust and significant induction of *CXCL10* mRNA when treated with IFN‐γ, and IFN‐γ combined with LPS (Figure S3A, S3B). In contrast to LPS and IFNγ, IL‐4 treatment did not change the expression of *TNFα, IL1β*, or *CXCL10*. Taken together, these data validate the responsiveness of iMG to established pro‐inflammatory signals.

To confirm our iMG are responsive to IL‐4 stimulation, we measured the mRNA levels of the nine known Interferon regulatory factors (IRFs), which are transcription factors and known regulators of microglia differentiation and transcriptional states.^29^, ^30^ Consistent with previous studies in primary microglia and bone marrow derived macrophages, IL‐4 treatment significantly increased *IRF4* expression (Figure S3C).^31^ IL‐4 treatment also led to a significant reduction in *IRF8* and *IRF9* RNA (Figure S3C). These data collectively demonstrate iMG are cytokine responsive and capable of selectively entering diverse transcriptional states.

### 3.4 iMG differentially respond to myelin debris and *S. aureus* bioparticles

**FIGURE 3:**
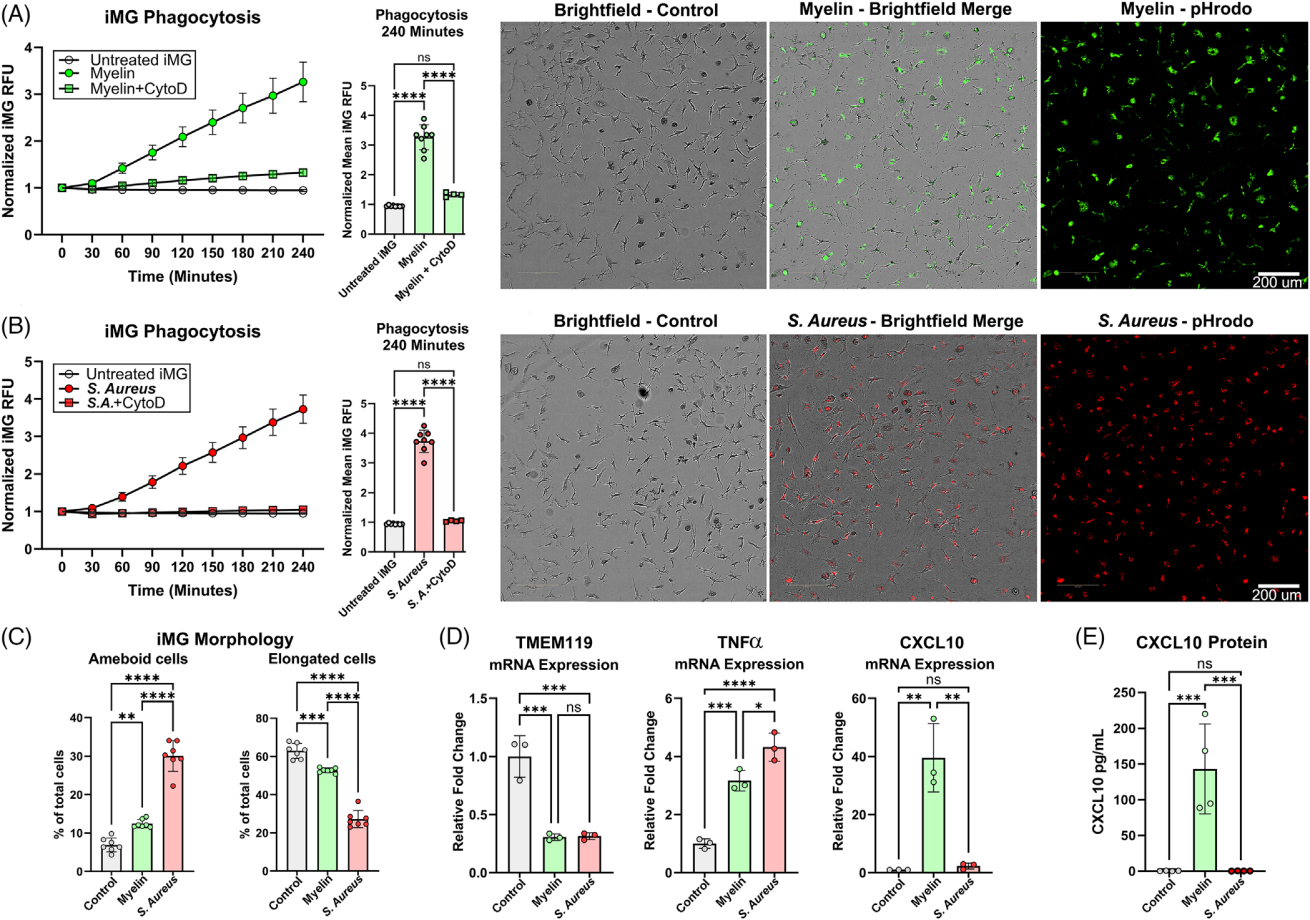
iMG are highly phagocytic and exhibit stimuli specific morphological and transcriptional responses. (A) Mean iMG fluorescence ± S.D is graphed over a 4‐h myelin debris phagocytosis assay. Cytochalasin D (CytoD) is used to inhibit phagocytosis. Mean iMG (≥700 iMG cells/well) fluorescence ± SD. Data are graphed at 4 h and compared by one‐way ANOVA. ****p ≤ 0.0001 with post‐hoc Tukey test. Representative pictures of live iMG with are shown 4 h after pHrodomyelin treatment. Scale bars 200 µm. (B) Mean iMG fluorescence ± SD is graphed over a 4‐h *S. aureus* phagocytosis assay. Mean iMG (≥700 iMG cells/well) fluorescence ± SD. at 4 h and compared by one‐way ANOVA. ****p ≤ 0.0001 with post‐hoc Tukey test. Representative pictures of live iMG with are shown 4 h after *S. aureus* treatment. Scale bars 200 µm. (C) Morphology of iMG cells is measured in response to myelin debris and *S. aureus* 4 h after treatments and compared by one‐way ANOVA. Data are represented as mean ± SD. **p ≤ 0.01; ****p ≤ 0.0001 with post‐hoc Tukey test. (D) Relative levels of *TNFα, TMEM119*, and *CXCL10* mRNA are measured in iMG after 24‐h treatment with myelin debris or *S. aureus* by qPCR with *RPL13* used as a reference gene. Data are represented as mean ± SD. **p ≤ 0.01; ****p ≤ 0.0001 with post‐hoc Tukey test. (E) CXCL10 secretion from iMG was measured by proximity ligation assay and compared by one‐way ANOVA. Data are represented as mean ± SD. ***p ≤ 0.001 with post‐hoc Tukey test.

Microglia are known as “professional phagocytes” and an established function of microglial cells in the central nervous system is the phagocytic clearance of myelin debris during aging and demyelinating disease.^32^ Recently, we developed a microglial myelin phagocytosis assay that tracks the cellular uptake of myelin debris labeled with the pH sensitive fluorescent dye pHrodo.^16^ We tested iMG for their ability to phagocytose pHrodomyelin debris in a 4 h phagocytosis assay and quantified cellular pHrodomyelin uptake by high‐content imaging (Figure 3A). We found iMG rapidly phagocytose pHrodomyelin debris and this process was significantly inhibited by treatment with cytochalasin D (Figure 3A). iMG were also treated with pHrodo labeled *S. aureus* bioparticles as an alternative to myelin. iMG readily phagocytosed these *S. aureus* bioparticles and this process was significantly inhibited by cytochalasin D (Figure 3B). Collectively, these data demonstrate that iMG are highly phagocytic toward diverse substrates in their culture environment in a similar manner to primary derived microglial cultures.

While performing the phagocytosis assays with myelin debris and *S. aureus* bioparticles, we observed that iMG were differentially shifting their cellular morphology in response to each stimulus. Changes in microglia shape are a well‐established feature of microglial biology in vivo as the cells survey and react to their environment.^33^ The appearance of spherical, ameboid microglia, as opposed to branched, elongated or ramified microglial cells, is associated with neuroinflammatory insults including traumatic brain injury and amyloid plaques. We quantified iMG morphology after 4 h of stimulation with these contrasting stimuli and we found that myelin debris had modest but statistically significant effects on iMG morphology by slightly increasing the percentage of ameboid iMG and modestly reducing the percentage of elongated iMG relative to controls (Figure 3C). In contrast to the modest effects of myelin debris, *S. aureus* bioparticles significantly increased the percentage of ameboid cells relative to myelin treated and control iMGs, while also significantly reducing the number of elongated iMG cells.

Given the differential morphological effects induced by myelin debris and *S. aureus* bioparticles on iMG, we further assessed the relative inflammatory transcriptional profile induced in iMG by these stimuli. Cultures of iMG were challenged with myelin debris or *S. aureus* bioparticles for 24 h and *TMEM119, TNFα* and *CXCL10* mRNA was measured by qPCR (Figure 3D). Both myelin debris and *S. aureus* bioparticles decreased levels of *TMEM119* mRNA and increased expression of *TNFα* mRNA relative to control iMG, indicating a shift away from a homeostatic state toward a more proinflammatory one. *S. aureus* caused a statistically significant and larger increase in the expression of *TNFα* mRNA than myelin debris, further suggesting it is a more direct inflammatory stimulus. Overall, these data are consistent with previous studies of microglia in vivo, which demonstrate the downregulation of microglial *TMEM119* expression under inflammatory conditions such as brain injury or amyloid plaque deposition.^34^ Following the observation that IFN‐γ treatment induced a very potent upregulation of *CXCL10* mRNA in iMG, we further tested the expression of this chemokine and found that myelin debris treatment significantly increased *CXCL10* mRNA expression while *S. aureus* had no significant effect. These data are in agreement with recent findings demonstrating that microglial release of CXCL10 plays an important role in the recruitment of peripheral immune cells during myelin debris clearance, and suggests that iMG respond to myelin debris in a physiologically relevant manner.^35^ To further validate that CXCL10 is released by iMG in response to myelin debris, we measured the concentration of CXCL10 protein in the iMG conditioned media. In agreement with our RNA measurements, myelin debris, but not *S. aureus* treatment, caused a robust and significant secretion of CXCL10 into the cell culture media (Figure 3E).

Together, these data demonstrate iMG respond differentially to discrete phagocytosed stimuli. Myelin debris clearance is a component of normal brain physiology and myelin debris is not overtly inflammatory to iMG when acutely phagocytosed, as compared to *S. aureus* particles which induce rapid changes in microglial morphology. iMG transcription and morphological responses are similar to established reactive microglia phenotypes observed in vivo.

### 3.5 Myelin debris treated iMG significantly alter lipid metabolism and form intracellular lipid droplets

**FIGURE 4:**
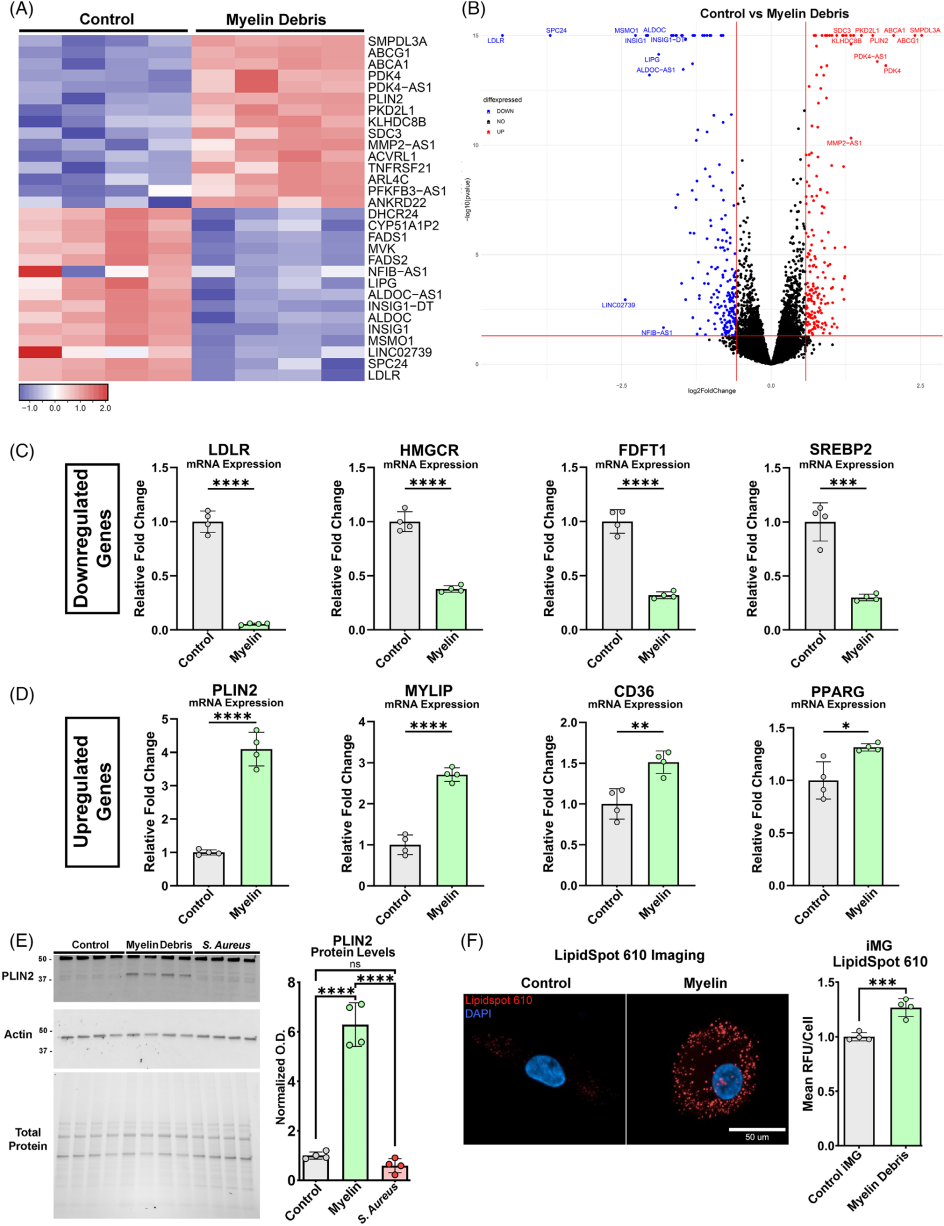
RNA sequencing reveals iMG significantly shift lipid metabolism in response to myelin debris. (A) The top 30 differentially expressed genes (DEGs) between control iMG and myelin debris stimulated iMG are graphed as a heat map. Each column is a biological replicate *n* = 4/treatment. (B) A volcano plot of DEGs in iMG in response to 24‐h myelin debris treatment is shown. (C) qPCR validation of downregulated genes identified by RNA sequencing in response to iMG myelin debris treatment. Data are represented as mean ± SD. ***p ≤ 0.001; ****p ≤ 0.0001 by unpaired *t*‐test. (D) qPCR validation of upregulated genes identified by RNA sequencing in iMG treated with myelin debris. Data are represented as mean ± SD. *p ≤ 0.05; **p ≤ 0.01; ****p ≤ 0.0001 by unpaired *t*‐test. (E) PLIN2 protein levels are measured in myelin debris and *S. aureus* bioparticle treated iMG by western blot with actin used as a loading control protein. Normalized optical density is graphed and data are graphed as mean ± SD. ****p ≤ 0.0001 by one‐way ANOVA with Tukey's post hoc test. (F) Lipid droplets were measured in iMG using LipidSpot 610 fluorescent dye. *t*‐test. Each data point is the average cellular fluorescence of at least 1800 iMG/well. Data are represented as mean ± SD. ***p ≤ 0.001 by unpaired *t*‐test, *n* = 4.

To examine potential effects of myelin debris on iMG transcriptional states, we treated iMG with myelin debris for 24 h and performed bulk RNA sequencing. Pathway analysis of differentially expressed genes (DEGs) between control iMG and myelin debris treated iMG revealed changes in many genes involved in lipid and cholesterol homeostasis. (Table S8—Myelin vs. control pathway analysis) (Table S9—Myelin vs. control DEGs). Further examination of differentially expressed genes revealed myelin debris treated iMG significantly upregulated lipid efflux transporters including ABCG1 and ABCA1, along with the lipid droplet associated gene PLIN2. (Figures 4A,B). SMPDL3A, a gene involved in cGAS‐STING pathway inhibition, was among the most significantly induced genes in myelin debris treated iMG.^36^ Myelin treated iMG significantly downregulated several genes involved in cholesterol synthesis including the master regulator transcription factor of cholesterol biosynthesis SREBF2 along with several of the enzymes in the cholesterol synthesis pathway including MVK, HMGCR, MSMO1, and FDFT1 (Table S9—Myelin vs. control DEGs). LDLR, a cell surface receptor involved in lipid uptake, was significantly downregulated while MYLIP, a ubiquitin ligase that facilitates LDLR protein degradation was upregulated^37^ (Figures 4A,B). Interestingly, the AD risk factor gene BIN1 was also among the significantly downregulated genes in iMG following myelin debris treatment^38^ (Table S9—Myelin vs. control DEGs).

To validate these RNA sequencing results, we repeated myelin debris stimulations in iMG and collected RNA for qPCR of selected transcripts (Figure 4C,D). In agreement with our bulk RNA sequencing results, we found that mRNA levels of *LDLR, HMGCR, FDFT1*, and *SREBP2* were significantly downregulated in myelin debris treated iMG, while mRNA levels *PLIN2, MYLIP, CD36*, and *PPARG* were all significantly upregulated. These findings further demonstrate the reproducibility of iMG transcriptional responses to stimuli in independent experiments using different methods to quantify mRNA transcripts.

PLIN2 lipid droplet formation in myeloid cells is increased under diverse physiologic conditions including increased levels of myelin debris.^39^, ^40^ Increased lipid droplet formation in the brain may be relevant to AD pathogenesis, as the ApoE4 allele has been shown to increase microglial lipid droplet burden.^41^, ^42^ To confirm PLIN2 transcriptional differences result in changes in PLIN2 protein, we stimulated cultures of iMG with myelin debris or *S. aureus* bioparticles for 24 h and measured PLIN2 by western blot (Figure 4E). Interestingly, PLIN2 was only upregulated by myelin debris treatment while PLIN2 levels were not changed between *S. aureus* treated iMG and controls. Cellular lipid droplets can be imaged using fluorescent lipophilic dyes including LipidSpot. To validate the formation of intracellular lipid droplets in our iMG, we performed high content imaging of control and myelin debris treated iMG and observed a robust and significant increase in LipidSpot fluorescence in myelin debris treated iMG (Figure 4F).

Together, these results collectively reveal that myelin debris uptake by iMG results in a comprehensive cellular response to control lipid levels by sequestering excess myelin lipids in lipid droplets, shutting down cholesterol biosynthesis, reducing LDL receptor mediated lipid uptake, and promoting lipid efflux through the ABCA transporters. This lipid droplet associated PLIN2 response was not induced by *S. aureus* bioparticles despite their robust phagocytic uptake by iMG, demonstrating the selectivity of this biological response toward specific phagocytosed material.

### 3.6 IL‐4 treatment upregulates TREM2 and DAP12 levels in iMG but does not increase myelin debris phagocytosis

**FIGURE 5:**
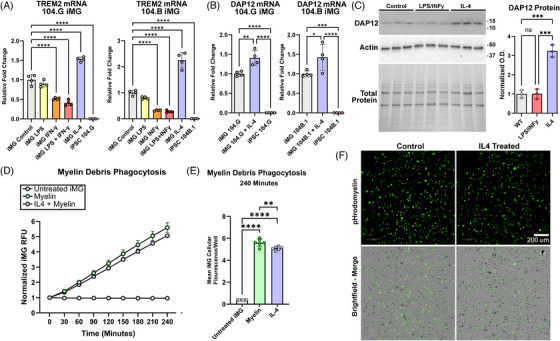
iMG express TREM2 and upregulate TREM2 in response to IL4, but phagocytosis of myelin debris is not increased after IL‐4 treatment. (A) TREM2 mRNA is measured in both IBRI 104.G and IBRI 104.B iMG in response to cytokine and LPS stimulation. The undifferentiated iPSC parent line is used as a negative control. Relative expression is graphed as mean ± SD. ***p ≤ 0.001 by one‐way ANOVA with Tukey's post hoc test. (B) DAP12 mRNA is measured in both IBRI 104.G and IBRI 104.B iMG in response to IL‐4 cytokine stimulation. Data are represented as mean ± SD. **p ≤ 0.01; ***p ≤ 0.001; ****p ≤ 0.0001 by unpaired *t*‐test. (C) DAP12 protein was measured in control, LPS+INFy, and IL‐4 treated iMG by western blot. Actin was used as a loading control. Normalized optical density is graphed and data are graphed as mean ± SD. ***p ≤ 0.001 by one‐way ANOVA with Tukey's post hoc test. (D) Control and IL‐4 treated iMG fluorescence ± S.D is graphed over a 4‐h pHrodomyelin debris phagocytosis assay. (E) Mean iMG (each dot represents ≥1800 iMG cells/well) pHrodomyelin fluorescence at 4 h ± SD. Data are graphed and compared by one‐way ANOVA. **p ≤ 0.01: ****p ≤ 0.0001 with post‐hoc Tukey test. Representative pictures of live iMG with are shown 4 h after pHrodomyelin treatment. Scale bars 200 µm.

TREM2 is an established AD risk gene richly expressed by microglia, and the TREM2 receptor has been implicated as having a functional role in phagocytosis.^43^ Noting that iMG are highly IL‐4 responsive (Figure S3C), and IL‐4 has been shown to upregulate TREM2 expression in macrophages,^44^ we measured TREM2 mRNA levels in IBRI 104.G and IBRI 104.B iMG in response to both inflammatory stimuli or IL‐4 for 24 h. TREM2 mRNA was not detected in undifferentiated iPSC lines but richly expressed in both IBRI 104.G and IBRI 104.B iMG cells. Interestingly, stimulation with TNFα inducing inflammatory stimuli like LPS and INFγ led to a reduced level of *TREM2* mRNA expression, while IL‐4 treatment caused significant increases in *TREM2* mRNA levels relative to control iMG (Figure 5A). TREM2 signals through the adapter DAP12, and like *TREM2, DAP12* mRNA levels in iMG were significantly increased in response to IL‐4 in both IBRI 104.G and IBRI 104.B derived iMG (Figure 5B). To confirm these changes were not limited to mRNA, we also measured DAP12 protein levels in iMG lysate by western blot and saw significantly increased DAP12 protein in IL‐4 treated samples. (Figure 5C).

TREM2 protein has roles in binding lipids and has been implicated as a phagocytic regulator.^43^ In a chronic demyelinating mouse model, TREM2 knockout microglia were found to have comparable uptake of myelin lipids compared to controls.^45^ We treated iMG with IL‐4 for 24 h to upregulate TREM2/DAP12 expression and measured pHrodomyelin phagocytosis. Phagocytosis of myelin debris was similar in control and IL‐4 treated iMG (Figures 5D–F). These data demonstrate TREM2 expression in iMG is dynamic, with inflammatory stimuli causing reduced TREM2 expression. IL‐4 cytokine treatment was sufficient to increase TREM2 and DAP12 levels, although this did not strongly affect acute myelin debris phagocytosis.

### 3.7 Stimulation of iMG with a TREM2 agonist antibody reduces acute myelin phagocytosis and drives chemokine expression in iMG

**FIGURE 6:**
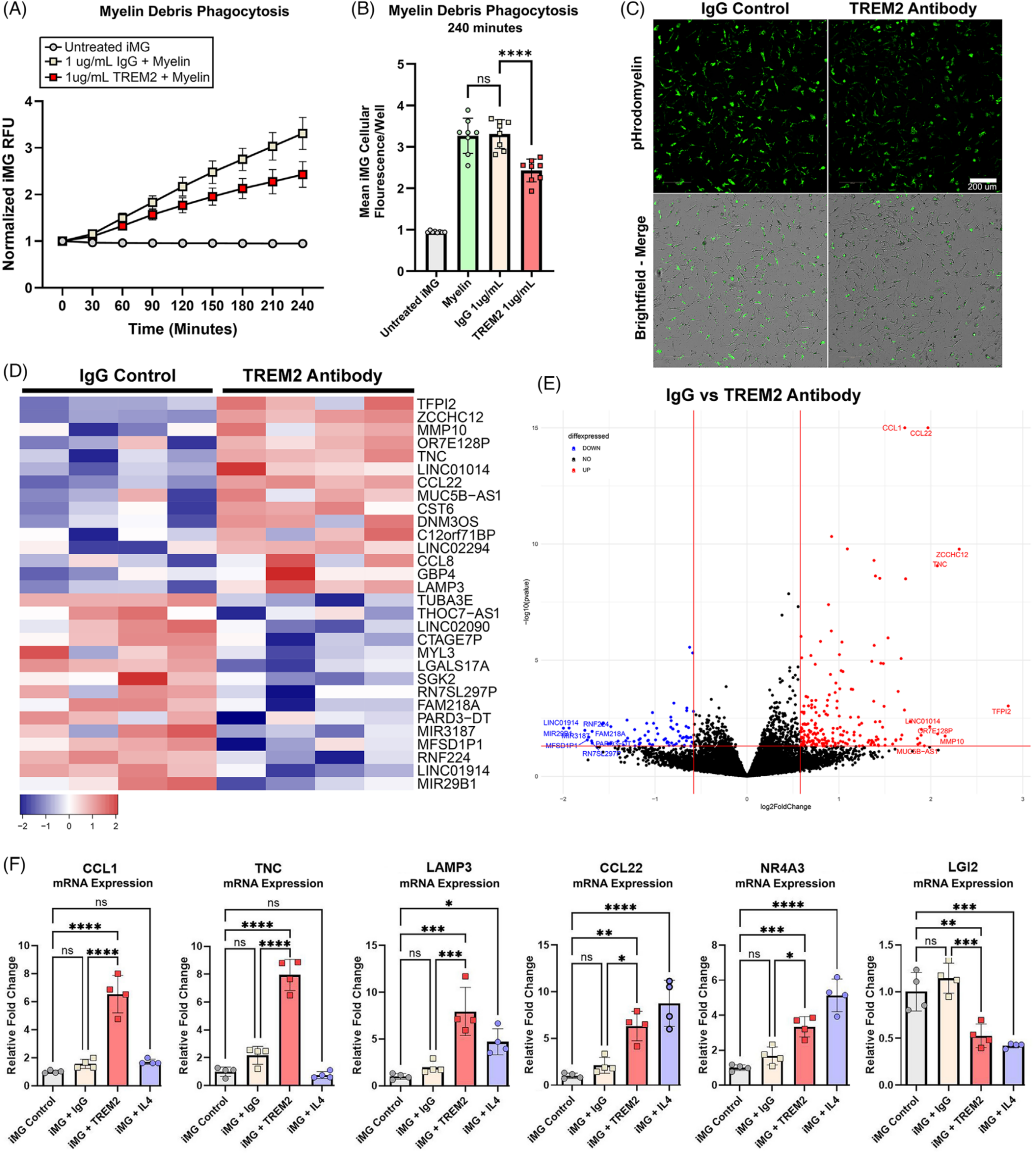
A TREM2 binding agonist antibody reduces pHrodomyelin debris phagocytosis but increases transcription of chemokine and lysosome genes. (A) IgG control and TREM2 antibody treated iMG fluorescence ± S.D is graphed over a 4‐h pHrodomyelin debris phagocytosis assay. (B) Mean iMG (each dot represents ≥700 iMG cells/well) pHrodomyelin fluorescence at 4 h ± SD. Data are graphed and compared by one‐way ANOVA. ****p ≤ 0.0001 with post‐hoc Tukey test. (C) Representative pictures of live iMG treated with IgG or TREM2 antibody are shown 4 h after pHrodomyelin treatment. Scale bars 200 µm. (D) The top 30 differentially expressed genes (DEGs) between IgG treated iMG and TREM2 antibody treated iMG are graphed as a heat map. Each column is a biological replicate *n* = 4/treatment. (E) A volcano plot of DEGs in iMG in response to 24‐h TREM2 antibody treatment is shown. (F) qPCR validation of differentially expressed genes identified by RNA sequencing of iMG treated with recombinant TREM2 antibody. Relative mRNA expression is measured in iMG after 24‐h treatment with IgG, TREM2 antibody and IL‐4. RPL13 is used as a reference gene. Data are represented as mean ± SD. *p ≤ 0.05; **p ≤ 0.01; ***p ≤ 0.01; ****p ≤ 0.0001 with post‐hoc Tukey test. *n* = 4/condition.

TREM2 targeting therapeutics for AD are under active investigation.^46^ We tested the responses of the IBRI 104.G iMG cells to a recombinant TREM2 agonist antibody engineered with LALAPG Fc insertions to abrogate binding to FcγRs.^17^, ^18^ We found that this TREM2 antibody clearly immunolabeled iMG significantly more than isotype control IgG in fluorescent imaging assays. (Figure S4). We examined the impact of this TREM2 antibody on iMG phagocytosis of myelin debris. iMG were treated with 1 µg/mL isotype control IgG or 1 µg/mL TREM2 agonist antibody and pHrodomyelin uptake was measured over 4 h by high‐content imaging. Interestingly, TREM2 antibody treatment significantly reduced myelin debris uptake compared to IgG control treatment (Figures 6A–C). As expected, IgG isotype control antibody did not significantly affect myelin phagocytosis when compared with untreated iMG (Figure 6B).

Given the specificity of the iMG response to myelin debris described earlier, we examined the transcriptional response induced by the recombinant TREM2 antibody. iMG were treated with TREM2 agonist antibody or isotype control IgG for 24 h and RNA was collected for sequencing. While the transcriptional response induced in iMG by the TREM2 antibody was much more modest than that induced by myelin debris, a specific and significant set of DEGs was still identified (Figure 6D,E) (Table S10—TREM2 Antibody_vs_IgG_DEGs). Among these genes included significant upregulation of chemokines including CCL8 and CCL22, along with the chemotactic extracellular matrix protein tenascin‐C (TNC). The lysosomal protein LAMP3, matrix metalloprotease 10 (MMP10), and nuclear receptor NR4A3 were also significantly upregulated.

To validate the reproducibility of these sequencing results, we treated IBRI 104.G iMG with isotype IgG or TREM2 antibody for 24 h. Following our observation that iMG respond to IL‐4 by increasing TREM2 levels, we also treated iMG with IL‐4 as a comparator against the TREM2 agonist antibody. We then performed qPCR to measure select transcripts of DEGs identified by bulk sequencing. Interestingly, *CCL1* and *TNC* were significantly induced by TREM2 antibody treatment but not IL‐4. *LAMP3, CCL22* and *NR4A3* were significantly increased by both TREM2 and IL‐4 treatment, while *LGI2* was downregulated by both TREM2 antibody and IL‐4 (Figure 6F). As expected, isotype IgG did not affect the mRNA expression of any of these select genes. These results demonstrate some transcriptional similarity in the iMG response to both TREM2 antibody and IL‐4 cytokine treatment. However, significant differences were also identified with the TREM2 antibody also driving unique transcriptional responses. Together, these results demonstrate an application of iMG in therapeutic molecule testing, which revealed informative and reproducible transcriptional readouts in response to TREM2 agonism.

## 4 DISCUSSION

Microglia are a highly specialized resident macrophage‐like cell population of the central nervous system that monitor the brain microenvironment to perform neuroprotective functions including the phagocytic clearance of cellular debris. Substantial evidence suggests that microglia play a central role in the development of late‐onset AD,^47^ and it is increasingly recognized that microglia also play a critical role in mediating the therapeutic effects of anti‐amyloid antibodies.^48^, ^49^ While there remains considerable interest in harnessing the neuroprotective potential of microglia to confer resilience to AD, it remains challenging to test therapeutic molecules in physiologically relevant model systems. Here we report the differentiation of iMG from newly developed human iPS cell lines generated by episomal reprogramming of erythroid progenitor cells cultured from blood PBMCs.

This protocol is based on an integration‐free method of reprogramming donor somatic cells to iPS cells with a high efficiency due to the use of p53 suppression by TP53 shRNA and nontransforming L‐Myc to enhance iPSC generation.^50^, ^51^ Episomal reprogramming using oriP/EBNA1 vectors allow for genetic footprint‐free reprogramming.^52^ Using this method, we generated two iPSC lines from donor PBMCs—IBRI 104.G and IBRI 104.B. These cell lines were characterized by immunofluorescence labeling of transcription factors and surface antigens that are typically highly expressed in PSCs and ESCs. Notably, we identify the expression of TRA‐1‐60, a transcription factor that is not present in the episomal reprogramming vectors, suggesting successful reprogramming of the donor somatic cells to iPSCs. Additionally, embryoid body assays were performed to confirm the pluripotent status of each iPS cell line. Immunofluorescence imaging of germ‐layer specific proteins and transcription factors identified the presence of mesoderm (vimentin), endoderm (SOX17), and ectoderm (β‐III‐tubulin).^53^, ^54^ Both iPSC lines were differentiated to iMG based on previously published protocols of cell fate induction using small molecules and human recombinant proteins, as shown in Figure 2. Several studies concerning the developmental trajectory of microglia have identified key cytokines, transcription factors, and cell receptors that can be utilized to recapitulate the development of these cells in vitro using human iPSCs.^11^, ^26^, ^55^, ^56^, ^57^, ^58^, ^59^, ^60^, ^61^ The stepwise iMG culture method described here builds on previously published protocols to generate iHPCs resembling the ontogeny of microglia with modifications to optimize their differentiation.^14^, ^25^

Many groups have identified methods to differentiate microglia‐like cells from human iPSCs to understand the cellular mechanisms underlying neurological diseases and injury using experimental assays and genetic editing.^15^, ^24^, ^62^, ^63^, ^64^, ^65^ These methods have enabled sensitive platforms to investigate the biology of human microglia; however, they often require labor intensive steps such as the generation of embryoid bodies for mesoderm induction and cell sorting, or depend on proprietary commercialized kits. The method described here provides a simplified monolayer differentiation method to generate iMG with fully defined experimental conditions, and the well‐defined reagents can readily be modified for further optimization, such as the addition or removal of factors to modulate WNT‐signaling during the mesoderm specification stage.

iMG generated using this method display similar characteristics to human microglia, including the expression of several established human microglial identity markers such as IBA1, TMEM119, P2Y12R, PU.1, TREM2, and HEXB. These iMG exhibit robust responses to diverse inflammatory stimuli by upregulating the transcription of relevant genes including TNFα, IL1β, and CXCL10. Notably, these iMG are morphologically dynamic and are maintained in culture as ramified or elongated cells under homeostatic like conditions and can rapidly change shape to become rounded or ameboid when stimulated acutely with potent inflammatory stimuli such as *S. aureus* bioparticles. iMG are also highly responsive to IL‐4 cytokine treatment and upregulate IRF4 while downregulating IRF8, which is notable as IRF8 plays a critical role in regulating the microglial transcriptional state and its disruption has been shown to induce AD associated transcriptional signatures.^30^ IL‐4 treatment also significantly increased TREM2 and DAP12 expression in iMG, which has also been observed in other myeloid cell types.^44^ IL‐4 may be a useful stimulant in iMG studies of TREM2 and its associated biology.

Microglial clearance of myelin debris is an established component of demyelinating brain injuries and plays a critical role in protecting brain health.^32^ In this study, we utilize myelin debris to characterize iMG in a variety of assays. iMG were treated with myelin debris in acute phagocytosis assays and also treated with myelin debris for 24 h to model microglial responses during chronic demyelinating disease. iMG rapidly phagocytosed pHrodomyelin debris in a process that was tracked and quantified by live cell high‐content imaging. iMG retained a ramified or branched and elongated cell morphology during acute myelin phagocytosis assays, suggesting myelin debris is not as inflammatory as *S. aureus* particles and that iMG respond differentially to phagocytosed materials. Microglia have been shown to produce chemokines such as CXCL10 in response to myelin debris in vivo, which serves to recruit peripheral immune cells which aid in the brain healing process.^35^ In this study, treatment of iMG with myelin debris induced chemokine responses including CXCL10 release, demonstrating iMG can respond to neurodegenerative stimuli in specific and biologically relevant ways.

Microglia are challenged with internalized lipids during phagocytosis and can accumulate intracellular lipid droplets during aging that are associated with impaired phagocytic function and inflammation.^42^, ^66^, ^67^ Moreover, the late‐onset AD risk gene PICALM was recently found to promote the formation of microglial lipid droplets while impairing phagocytosis.^68^ We found that treatment of iMG with myelin debris had potent effects on the transcription of several lipid homeostasis genes. Cholesterol synthesis genes including the master transcriptional regulator SREBF2 and cholesterol synthesis enzymes including HMGCR, MVK and FDFT1 were downregulated. Cholesterol efflux transporters including ABCA1, ABCD1, and ABCG1 were upregulated, while expression of LDL receptor was reduced. Myelin treated iMG also increased expression of MYLIP, a ubiquitin ligase involved in LDLR protein degradation.^69^ Analysis of the top differentially expressed genes also revealed that myelin treated iMG strongly and significantly upregulated SMPDL3A (*p*‐adjusted value = 1.33e‐54), which is induced by LXR and inhibits the cGAS‐STING immune response pathway by degrading cGAMP.^36^ This transcriptional profiling revealed a unique cellular mechanism coupling lipid metabolism to immune modulation suggesting that microglia engage specific cellular responses during myelin phagocytosis to limit inflammation and neurotoxicity.

Interestingly, myelin lipids accumulated in microglia, as myelin treatment of iMG also induced expression of genes involved in lipid droplet formation including PPARG and PLIN2, and increased iMG droplet content in cellular imaging assays. This myelin debris induced iMG response contrasts to the reaction induced by *S. aureus* bioparticles which did not increase the levels of the lipid droplet protein PLIN2. Consistent with previous studies in mouse microglia and macrophages, human iMG treated with myelin also upregulated the scavenger receptor CD36 which has been shown to play a critical role in myelin phagocytosis and suppression of myelin‐induced inflammatory responses.^70^ The AD risk gene BIN1, which has been implicated as a regulator of microglial immune functions, was also identified as a significantly downregulated (*p* adjusted value = 0.03) gene in myelin treated iMG.^70^ Altogether, this transcriptional response by iMG to myelin debris reveals a comprehensive and specific cellular response to control lipid intake, production, trafficking, and storage while also regulating immune signaling. These data support the use of our iMG as a model system to study transcriptional responses to neurodegeneration‐associated stimuli and further suggest myelin debris serves as a useful stimulus to burden microglia with increased lipids.

Microglial targeted therapeutics are an enduring goal of translational glial cell research; however, recent clinical trials targeting microglial TREM2 with antibodies have failed to meet their primary endpoints.^46^ Still, TREM2 remains an active target of investigation, and alternative molecular approaches to modulate the signaling and functioning of TREM2 have shown benefits in transgenic animal model systems.^71^, ^72^ Here we evaluated iMG as a platform to study emerging microglial targeting therapeutic molecules by generating a recombinant TREM2 agonist antibody from published sequences and studying the transcriptional and functional responses of iMG to this molecule. While previous research has demonstrated TREM2 knockout microglia have impaired phagocytosis and that TREM2 receptor itself may directly bind lipids, it remains uncertain if TREM2 directly participates in the process of microglial phagocytosis. Notably, TREM2 knockout mouse microglia have been shown to have comparable uptake of myelin lipids in an acute demyelination mouse model in vivo.^45^ We found that IL‐4 mediated upregulation of TREM2 did not enhance myelin phagocytosis, while acute treatment of iMG with the TREM2 antibody significantly impaired myelin debris uptake. These results agree with a rigorous study in which treatment of a demyelinating mouse model with TREM2 agonist antibody led to increased myelin debris accumulation in the brain and impaired lesion resolution.^73^ TREM2 agonist antibodies similar to the antibody used in this study have been found to induce TREM2 protein internalization,^74^ potentially contributing to the impaired myelin phagocytosis we observe in TREM2 antibody treated iMG.

TREM2 likely plays a critical role in brain healing of demyelinating lesions as blocking TREM2 with an antibody impairs myelin repair.^75^ Our results collectively suggest TREM2 targeting antibodies do not enhance uptake of myelin but instead induce chemokine and lysosomal responses in iMG. It remains to be studied how iMG polarized toward alternative transcriptional or activation states may respond toward similar TREM2 targeting molecules.

As part of the TREAT‐AD Center's mission to develop robust methods, our iPSC generation and iMG differentiation protocols reproducibly generate microglia‐like cells that respond consistently and specifically to diverse stimuli and pharmacological agents. These iPSC lines and iMG platform serve as a valuable approach to inform and supplement drug lead optimization strategies and potentially reveal unexpected responses of human microglia‐like cells to potential therapeutics. Future research will expand on this model system by utilizing cells in complex multicellular culture models and exploring genetic editing approaches to generate AD‐relevant microglial cell lines.

## AUTHOR CONTRIBUTIONS

Angela K. Haskell, Joshua A. Kulas, Louis F. Stancato, Timothy I. Richardson and Abdul Qadir Syed conceptualized this study. Angela K. Haskell, Joshua A. Kulas, William E. Carter, June Javens‐Wolfe. Jacob S. Smiley, Shaoyou Chu, Mustapha Moussaif and Raven Dance Hinkel developed methodology. Investigation was led by Angela K. Haskell, Joshua A. Kulas, William E. Carter, Raven Dance Hinkel, Jacob S. Smiley. June Javens‐Wolfe and Mustapha Moussaif. Data analysis was performed by Angela K. Haskell, Joshua A. Kulas, Travis Johnson, Olivia Lazaro, Sylvia Robertson and June Javens‐Wolfe. Funding was acquired by Alan D. Palkowitz, Timothy I. Richardson and Bruce T. Lamb to support this project. Angela K. Haskell, Joshua A. Kulas, Abdul Qadir Syed and Louis F. Stancato supervised this study. Angela K. Haskell and Joshua A. Kulas drafted this manuscript with review and edits from Abdul Qadir Syed, Louis F. Stancato, Timothy I. Richardson, Shaoyou Chu, Alan D. Palkowitz, Jeffrey L. Dage and Bruce T. Lamb.

## CONFLICT OF INTEREST STATEMENT

Jeffrey L. Dage (JLD) is an inventor on patents or patent applications assigned to Eli Lilly and Company relating to the assays, methods, reagents and / or compositions of matter for P‐tau assays and Aβ targeting therapeutics. JLD has/is served/serving as a consultant or on advisory boards for Eisai, Abbvie, Genotix Biotechnologies Inc, Gates Ventures, Syndeio Biosciences, Dolby Family Ventures, Karuna Therapeutics, Alzheimer's Disease Drug Discovery Foundation, AlzPath Inc., Cognito Therapeutics, Inc., Eli Lilly and Company, Prevail Therapeutics, Neurogen Biomarking, Spear Bio, Rush University, University of Kentucky, Tymora Analytical Operations, MindImmune Therapeutics, Inc, Early is Good, and Quanterix. JLD has received research support from ADx Neurosciences, Fujirebio, Roche Diagnostics and Eli Lilly and Company in the past two years. JLD has received speaker fees from Eli Lilly and Company and LabCorp. JLD is a founder and advisor for Monument Biosciences and Dage Scientific LLC. JLD has stock or stock options in Eli Lilly and Company, Genotix Biotechnologies, MindImmune Therapeutics Inc., AlzPath Inc., Neurogen Biomarking, and Monument Biosciences. Jeffrey L. Dage, Timothy I. Richardson, Bruce T. Lamb, and Alan Palkowitz are founders and consultants for Monument Biosciences. All funding provided to the institution and individual authors has been disclosed in the funding information and the declaration of interest section. No other authors have conflict of interests to disclose. Author disclosures are available in the Supporting Information.

## CONSENT STATEMENT

The blood samples to isolate PBMCs and reprogrammed to generate iPSC lines used in this study were obtained from BioIVT (catalog no. HUMANWBK2‐0000399) from IRB‐consented donors by the commercial vendor prior to distribution. BioIVT confirms that the samples are de‐identified and that the informed consent process was handled by the vendor in accordance with applicable ethical and regulatory standards.

## Supporting information



Supporting Information

Supporting Information

Supporting Information

Supporting Information

Supporting Information

## References

[1] Ginhoux F , Greter M , Leboeuf M , et al. Fate mapping analysis reveals that adult microglia derive from primitive macrophages. Science. 2010;330(6005):841–845. 20966214 10.1126/science.1194637 PMC3719181

[2] Prinz M , Priller J , Sisodia SS , Ransohoff RM . Heterogeneity of CNS myeloid cells and their roles in neurodegeneration. Nat Neurosci. 2011;14(10):1227–1235. 21952260 10.1038/nn.2923

[3] Eskandari‐Sedighi G , Crichton M , Zia S , et al. Alzheimer's disease associated isoforms of human CD33 distinctively modulate microglial cell responses in 5XFAD mice. Mol Neurodegener. 2024;19(1):42. 38802940 10.1186/s13024-024-00734-8 PMC11129479

[4] Wang Y , Ulland TK , Ulrich JD , et al. TREM2‐mediated early microglial response limits diffusion and toxicity of amyloid plaques. J Exp Med. 2016;213(5):667–675. 27091843 10.1084/jem.20151948 PMC4854736

[5] Ennerfelt H , Frost EL , Shapiro DA , et al. SYK coordinates neuroprotective microglial responses in neurodegenerative disease. Cell. 2022;185(22):4135–4152. e22. 36257314 10.1016/j.cell.2022.09.030 PMC9617784

[6] Ennerfelt H , Holliday C , Shapiro D,etal . CARD9 attenuates Aβ pathology and modifies microglial responses in an Alzheimer's disease mouse model. Proc Natl Acad Sci U S A. 2023;120(24):e2303760120. 37276426 10.1073/pnas.2303760120 PMC10268238

[7] Messenger EJ , Baar SA , Bedford LM , et al. PLCG2 modulates TREM2 expression and signaling in response to Alzheimer's disease pathology. Alzheimers Dement. 2025;21(5):e70231. 40346446 10.1002/alz.70231 PMC12064341

[8] Lin PB , Tsai AP , Soni D , et al. INPP5D deficiency attenuates amyloid pathology in a mouse model of Alzheimer's disease. Alzheimers Dement. 2023;19(6):2528–2537. 36524682 10.1002/alz.12849

[9] Spangenberg E , Severson PL , Hohsfield LA , et al. Sustained microglial depletion with CSF1R inhibitor impairs parenchymal plaque development in an Alzheimer's disease model. Nat Commun. 2019;10(1):3758. 31434879 10.1038/s41467-019-11674-z PMC6704256

[10] Bang J , Yoo Y . Rationale and emerging evidence for microglial replacement in Alzheimer's disease. Mol Cells. 2025:100265. 40818647 10.1016/j.mocell.2025.100265 PMC12444172

[11] Butovsky O , Jedrychowski MP , Moore CS , et al. Identification of a unique TGF‐β‐dependent molecular and functional signature in microglia. Nat Neurosci. 2014;17(1):131–143. 24316888 10.1038/nn.3599 PMC4066672

[12] Woolf Z , Stevenson TJ , Lee K , et al. In vitro models of microglia: a comparative study. Sci Rep. 2025;15(1):15621. 40320508 10.1038/s41598-025-99867-z PMC12050316

[13] Fumagalli L , Nazlie Mohebiany A , Premereur J , et al. Microglia heterogeneity, modeling and cell‐state annotation in development and neurodegeneration. Nat Neurosci. 2025;28(7):1381–1392. 40195564 10.1038/s41593-025-01931-4

[14] Cao X , van den Hil FE , Mummery CL , Orlova VV . Generation and Functional Characterization of Monocytes and Macrophages Derived from Human Induced Pluripotent Stem Cells. Curr Protoc Stem Cell Biol. 2020;52(1):e108. 32159928 10.1002/cpsc.108 PMC7154707

[15] McQuade A , Blurton‐Jones M . Human Induced Pluripotent Stem Cell‐Derived Microglia (hiPSC‐Microglia). Methods Mol Biol. 2022;2454:473–482. 34773245 10.1007/7651_2021_429

[16] Mason E , Soni D , Chu S . Microglial phagocytosis/Cell health high‐content assay. Curr Protoc. 2023;3(3):e724. 36971657 10.1002/cpz1.724 PMC10433541

[17] Kate Monroe TS , Avogadri‐Connors F , Tassi I , Lam H , Rosenthol A . Anti‐Trem2 Antibodies and Methods and Use Thereof. United States; 2019. U.S.P.a.T. Office, Editor.

[18] Wang X , Mathieu M , Brezski RJ . IgG Fc engineering to modulate antibody effector functions. Protein Cell. 2018;9(1):63–73. 28986820 10.1007/s13238-017-0473-8 PMC5777978

[19] Love M , Huber W , Anders S . Moderated estimation of fold change and dispersion for RNA‐seq data with DESeq2. Genome Biol. 2014;15(12):550. 25516281 10.1186/s13059-014-0550-8 PMC4302049

[20] Wickham H . ggplot2: Elegant Graphics for Data Analysis. Springer‐Verlag; 2016.

[21] Zhao S , Guo Y , Sheng Q , Shyr Y . Heatmap3: an improved heatmap package with more powerful and convenient features. BMC Bioinformatics. 2014;15(Suppl 10):P16. doi:10.1186/1471-2105-15-S10-P16

[22] Wu T , Hu E , Xu S , et al. clusterProfiler 4.0: a universal enrichment tool for interpreting omics data. Innovation. 2021;2(3):100141. 34557778 10.1016/j.xinn.2021.100141 PMC8454663

[23] Yu G , He Q . ReactomePA: an R/Bioconductor package for reactome pathway analysis and visualization. Mol Biosyst. 2016;12(2):477–479. 26661513 10.1039/c5mb00663e

[24] Abud EM , Ramirez RN , Martinez ES , et al. iPSC‐derived human microglia‐like cells to study neurological diseases. Neuron. 2017;94(2):278–293.e9. 28426964 10.1016/j.neuron.2017.03.042 PMC5482419

[25] McQuade A , Coburn M , Tu CH , Hasselmann J , Davtyan H , Blurton‐Jones M . Development and validation of a simplified method to generate human microglia from pluripotent stem cells. Mol Neurodegener. 2018;13(1):67. 30577865 10.1186/s13024-018-0297-x PMC6303871

[26] Wang Y , Szretter KJ , Vermi W , et al. IL‐34 is a tissue‐restricted ligand of CSF1R required for the development of Langerhans cells and microglia. Nat Immunol. 2012;13(8):753–760. 22729249 10.1038/ni.2360 PMC3941469

[27] Dadwal S , Heneka M . Microglia heterogeneity in health and disease. FEBS Open Bio. 2024;14(2):217–229. 10.1002/2211-5463.13735 PMC10839410 37945346

[28] Allen J . IL‐4 and IL‐13: regulators and Effectors of Wound Repair. Annu Rev Immunol. 2023;41:229–254. 36737597 10.1146/annurev-immunol-101921-041206

[29] Minten C , Terry R , Deffrasnes C , King NJC , Campbell IL . IFN regulatory factor 8 is a key constitutive determinant of the morphological and molecular properties of microglia in the CNS. PLoS One. 2012;7(11):e49851. 23166780 10.1371/journal.pone.0049851 PMC3498170

[30] Saeki K , Pan R , Lee E , Kurotaki D , Ozato K . IRF8 defines the epigenetic landscape in postnatal microglia, thereby directing their transcriptome programs. Nat Immunol. 2024;25(10):1928–1942. 39313544 10.1038/s41590-024-01962-2

[31] El Chartouni C , Schwarzfischer L , Rehli M . Interleukin‐4 induced interferon regulatory factor (Irf) 4 participates in the regulation of alternative macrophage priming. Immunobiology. 2010;215(9‐10):821–825. 20580461 10.1016/j.imbio.2010.05.031

[32] Gao R , Song S , Tian M , Wang L , Zhang Y , Li X . Myelin debris phagocytosis in demyelinating disease. Glia. 2024;72(11):1934–1954. 39073200 10.1002/glia.24602

[33] Vidal‐Itriago A , Radford RAW , Aramideh JA , et al. Microglia morphophysiological diversity and its implications for the CNS. Front Immunol. 2022;13:997786. 36341385 10.3389/fimmu.2022.997786 PMC9627549

[34] Ruan C , Elyaman W . A new understanding of TMEM119 as a marker of microglia. Front Cell Neurosci. 2022;16:902372. 35769325 10.3389/fncel.2022.902372 PMC9234454

[35] Groh J , Feng R , Yuan X , et al. Microglia activation orchestrates CXCL10‐mediated CD8(+) T cell recruitment to promote aging‐related white matter degeneration. Nat Neurosci. 2025;28(6):1160–1173. 40404995 10.1038/s41593-025-01955-w PMC12148934

[36] Hou Y , Wang Z , Liu P , et al. SMPDL3A is a cGAMP‐degrading enzyme induced by LXR‐mediated lipid metabolism to restrict cGAS‐STING DNA sensing. Immunity. 2023;56(11):2492–2507.e10. 37890481 10.1016/j.immuni.2023.10.001

[37] Zelcer N , Hong C , Boyadjian R , Tontonoz P . LXR regulates cholesterol uptake through Idol‐dependent ubiquitination of the LDL receptor. Science. 2009;325(5936):100–104. 19520913 10.1126/science.1168974 PMC2777523

[38] Seshadri S , Fitzpatrick AL , Ikram MA , et al. Genome‐wide analysis of genetic loci associated with Alzheimer disease. JAMA. 2010;303(18):1832–1840. 20460622 10.1001/jama.2010.574 PMC2989531

[39] Loix M , Wouters E , Vanherle S , et al. Perilipin‐2 limits remyelination by preventing lipid droplet degradation. Cell Mol Life Sci. 2022;79(10):515. 36100764 10.1007/s00018-022-04547-0 PMC11803036

[40] Madsen S , Delgado AC , Cadilhac C , et al. A fluorescent perilipin 2 knock‐in mouse model reveals a high abundance of lipid droplets in the developing and adult brain. Nat Commun. 2024;15(1):5489. 38942786 10.1038/s41467-024-49449-w PMC11213871

[41] Haney MS , Pálovics R , Munson CN , et al. APOE4/4 is linked to damaging lipid droplets in Alzheimer's disease microglia. Nature. 2024;628(8006):154–161. 38480892 10.1038/s41586-024-07185-7 PMC10990924

[42] Friday CM , Stephens IO , Smith CT , et al. APOE4 reshapes the lipid droplet proteome and modulates microglial inflammatory responses. Neurobiol Dis. 2025;212:106983. 40451545 10.1016/j.nbd.2025.106983 PMC12187248

[43] Colonna M . The biology of TREM receptors. Nat Rev Immunol. 2023;23(9):580–594. 36750615 10.1038/s41577-023-00837-1 PMC9904274

[44] Turnbull IR , Gilfillan S , Cella M , et al. Cutting edge: tREM‐2 attenuates macrophage activation. J Immunol. 2006;177(6):3520–3524. 16951310 10.4049/jimmunol.177.6.3520

[45] Nugent AA , Lin K , van Lengerich B , et al. TREM2 regulates microglial cholesterol metabolism upon chronic phagocytic challenge. Neuron. 2020;105(5):837–854.e9. 31902528 10.1016/j.neuron.2019.12.007

[46] Colonna M , Holtzman D . Rethinking TREM2 as a target for Alzheimer's disease after the INVOKE‐2 trial failure. Nat Med. 2025;31(10):3217–3218. 40603729 10.1038/s41591-025-03816-2

[47] Sudwarts A , Thinakaran G . Alzheimer's genes in microglia: a risk worth investigating. Mol Neurodegener. 2023;18(1):90. 37986179 10.1186/s13024-023-00679-4 PMC10662636

[48] Cadiz MP , Gibson KA , Todd KT , et al. Aducanumab anti‐amyloid immunotherapy induces sustained microglial and immune alterations. J Exp Med. 2024;221(2):e20231363. 38226975 10.1084/jem.20231363 PMC10791560

[49] van Olst L , Simonton B , Edwards AJ , et al. Microglial mechanisms drive amyloid‐β clearance in immunized patients with Alzheimer's disease. Nat Med. 2025;31(5):1604–1616. 40050704 10.1038/s41591-025-03574-1 PMC12092304

[50] Okita K , Matsumura Y , Sato Y , et al. A more efficient method to generate integration‐free human iPS cells. Nat Methods. 2011;8(5):409–412. 21460823 10.1038/nmeth.1591

[51] Kim D , Kim C , Moon J , et al. Generation of human induced pluripotent stem cells by direct delivery of reprogramming proteins. Cell Stem Cell. 2009;4(6):472–476. 19481515 10.1016/j.stem.2009.05.005 PMC2705327

[52] Malik N , Rao M . A review of the methods for human iPSC derivation. Methods Mol Biol. 2013;997:23–33. 23546745 10.1007/978-1-62703-348-0_3 PMC4176696

[53] Stenberg J , Elovsson M , Strehl R , Kilmare E , Hyllner J , Lindahl A . Sustained embryoid body formation and culture in a non‐laborious three dimensional culture system for human embryonic stem cells. Cytotechnology. 2011;63(3):227–237. 21409453 10.1007/s10616-011-9344-y PMC3081049

[54] Schroeder IS , Sulzbacher S , Nolden T , et al. Induction and selection of Sox17‐expressing endoderm cells generated from murine embryonic stem cells. Cells Tissues Organs. 2012;195(6):507–523. 22123608 10.1159/000329864

[55] Matcovitch‐Natan O , Winter DR , Giladi A , et al. Microglia development follows a stepwise program to regulate brain homeostasis. Science. 2016;353(6301):aad8670. 27338705 10.1126/science.aad8670

[56] Ginhoux F , Lim S , Hoeffel G , Low D , Huber T . Origin and differentiation of microglia. Front Cell Neurosci. 2013;7:45. 23616747 10.3389/fncel.2013.00045 PMC3627983

[57] Askew K , Li K , Olmos‐Alonso A , et al. Coupled proliferation and apoptosis maintain the rapid turnover of microglia in the adult brain. Cell Rep. 2017;18(2):391–405. 28076784 10.1016/j.celrep.2016.12.041 PMC5263237

[58] Kierdorf K , Erny D , Goldmann T , et al. Microglia emerge from erythromyeloid precursors via Pu.1‐ and Irf8‐dependent pathways. Nat Neurosci. 2013;16(3):273–280. 23334579 10.1038/nn.3318

[59] Réu P , Khosravi A , Bernard S , et al. The lifespan and turnover of microglia in the human brain. Cell Rep. 2017;20(4):779–784. 28746864 10.1016/j.celrep.2017.07.004 PMC5540680

[60] Saederup N , Cardona AE , Croft K , et al. Selective chemokine receptor usage by central nervous system myeloid cells in CCR2‐red fluorescent protein knock‐in mice. PLoS One. 2010;5(10):e13693. 21060874 10.1371/journal.pone.0013693 PMC2965160

[61] Sharma R , Mei A , Mathew V , Kashpur O , Wallingford MC . Interaction of extraembryonic microglia and neonatal brain development. Exp Neurol. 2022;351:113986. 35065053 10.1016/j.expneurol.2022.113986

[62] Claes C , Van Den Daele J , Boon R , et al. Human stem cell‐derived monocytes and microglia‐like cells reveal impaired amyloid plaque clearance upon heterozygous or homozygous loss of TREM2. Alzheimers Dement. 2019;15(3):453–464. 30442540 10.1016/j.jalz.2018.09.006

[63] Muffat J , Li Y , Yuan B , et al. Efficient derivation of microglia‐like cells from human pluripotent stem cells. Nat Med. 2016;22(11):1358–1367. 27668937 10.1038/nm.4189 PMC5101156

[64] Reich M , Paris I , Ebeling M , et al. Alzheimer's risk gene TREM2 determines functional properties of new type of human iPSC‐derived microglia. Front Immunol. 2020;11:617860. 33613545 10.3389/fimmu.2020.617860 PMC7887311

[65] Vanhee S , De Mulder K , Van Caeneghem Y , et al. In vitro human embryonic stem cell hematopoiesis mimics MYB‐independent yolk sac hematopoiesis. Haematologica. 2015;100(2):157–166. 25381126 10.3324/haematol.2014.112144 PMC4803135

[66] Marschallinger J , Iram T , Zardeneta M , et al. Lipid‐droplet‐accumulating microglia represent a dysfunctional and proinflammatory state in the aging brain. Nat Neurosci. 2020;23(2):194–208. 31959936 10.1038/s41593-019-0566-1 PMC7595134

[67] Wu X , Miller JA , Lee BTK , Wang Y , Ruedl C . Reducing microglial lipid load enhances β amyloid phagocytosis in an Alzheimer's disease mouse model. Sci Adv. 2025;11(6):eadq6038. 39908361 10.1126/sciadv.adq6038 PMC11797491

[68] Kozlova A , Zhang S , Sudwarts A , et al. PICALM Alzheimer's risk allele causes aberrant lipid droplets in microglia. Nature. 2025;646(8087):1178–1186. 40903578 10.1038/s41586-025-09486-x PMC12571902

[69] Choi J , Gao J , Kim J , Hong C , Kim J , Tontonoz P . The E3 ubiquitin ligase Idol controls brain LDL receptor expression, ApoE clearance, and Aβ amyloidosis. Sci Transl Med. 2015;7(314):314ra184. 10.1126/scitranslmed.aad1904 PMC7153735 26582899

[70] Grajchen E , Wouters E , van de Haterd B , et al. CD36‐mediated uptake of myelin debris by macrophages and microglia reduces neuroinflammation. J Neuroinflammation. 2020;17(1):224. 32718316 10.1186/s12974-020-01899-x PMC7384221

[71] Kraller M , Faßbender J , Jabali A , et al. Novel fully human high‐affinity anti‐TREM2 antibody shows efficacy in clinically relevant Alzheimer´s mouse model. Alzheimers Res Ther. 2025;17(1):114. 40405265 10.1186/s13195-025-01759-x PMC12096560

[72] Schlepckow K , Morenas‐Rodríguez E , Hong S , Haass C . Stimulation of TREM2 with agonistic antibodies‐an emerging therapeutic option for Alzheimer's disease. Lancet Neurol. 2023;22(11):1048–1060. 37863592 10.1016/S1474-4422(23)00247-8

[73] Etxeberria A , Shen YA , Vito S , et al. Neutral or detrimental effects of TREM2 agonist antibodies in preclinical models of Alzheimer's disease and multiple sclerosis. J Neurosci. 2024;44(29):e2347232024. 38830764 10.1523/JNEUROSCI.2347-23.2024 PMC11255434

[74] Long H , Simmons A , Mayorga A , et al. Preclinical and first‐in‐human evaluation of AL002, a novel TREM2 agonistic antibody for Alzheimer's disease. Alzheimers Res Ther. 2024;16(1):235. 39444037 10.1186/s13195-024-01599-1 PMC11515656

[75] Hou J , Magliozzi R , Chen Y , et al. Acute TREM2 inhibition depletes MAFB‐high microglia and hinders remyelination. Proc Natl Acad Sci U S A. 2025;122(13):e2426786122. 40131948 10.1073/pnas.2426786122 PMC12002275

