# Supplementary material for: Generation and characterization of iPSC‐derived microglia for in vitro modeling of stimuli‐specific neuroimmune responses
Source: Alzheimers Dement. 2026 Feb 4;22(2):e71117. doi: 10.1002/alz.71117 (PMC12872402; doi:10.1002/alz.71117)
Supplement: Supplementary file 1 — Supporting Information [file ALZ-22-e71117-s005.docx]

**Figure S1. Characterization of the IBRI 104.B iPSC line.** (A) Fluorescent images of IBRI 104.B iPSC colonies stained with antibodies against TRA-1-60, NANOG, SSEA4 and OCT4, with a DAPI DNA counterstain. Scale bar, 100 um. (B) Karyotyping of the IBRI 104.B iPSC line. (C) IBRI 104.G iPSC cells were spontaneously differentiated into the primary germ layers stained with antibodies against Vimentin (mesoderm), SOX17 (endoderm), and β-III-Tubulin (ectoderm). Scale bar, 100 μm.

**Figure S2. Immunofluorescent imaging of iMG for microglial proteins** High-content imaging of iMG cultures (n=4 total cultures, ~2000 cells imaged / culture) was performed to quantify the percentage iMG fluorescent for each marker (A). Representative images of P2RY12R (B), PU.1 (C) and TREM2 (D) are shown with negative controls showing cellular fluorescence in the absence of each primary antibody. Scale bar 100 μm.


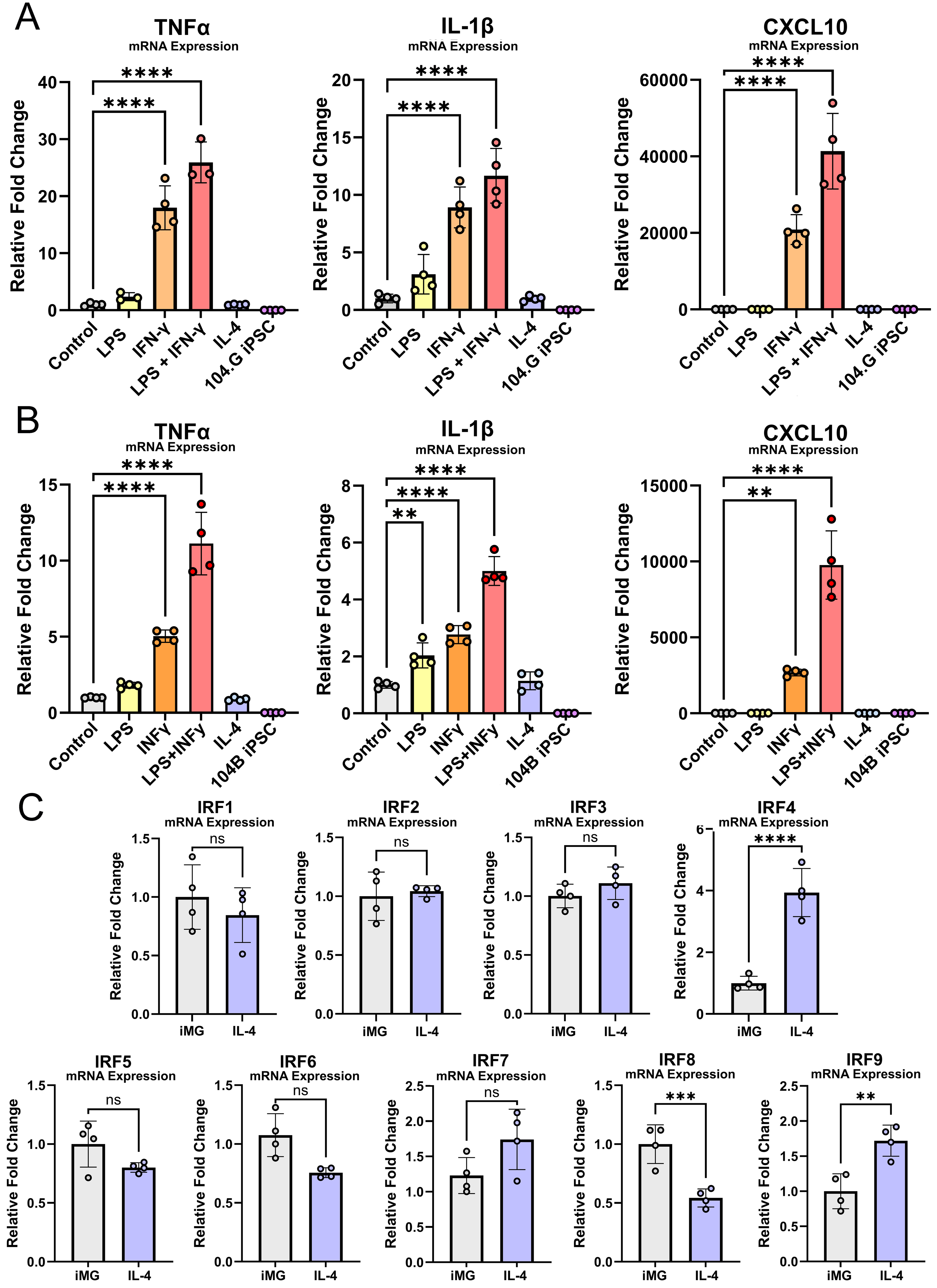


**Figure S3. iMG are responsive to cytokine and LPS stimulation.** (A) Relative *TNFα, IL-1β* and *CXCL10* mRNA levels are measured in IBRI 104.G iMG after 24 hour treatment with stimulants and compared by one-way ANOVA. The undifferentiated iPSC parent line is used as a negative control. Data are represented as mean ± SD. ****p≤0.0001 with post-hoc Tukey test. Only significant differences compared to controls are displayed. (B) Relative *TNFα, IL-1β* and *CXCL10* mRNA levels are measured in IBRI 104.B iMG after 24 hour treatment with stimulants and compared by one-way ANOVA. Data are represented as mean ± SD. **p≤0.01; ****p≤0.0001 with post-hoc Tukey test. Only significant differences compared to controls are displayed. (C) Relative mRNA levels of the IRF transcription factors are measured in IBRI 104.G iMG in response to 24 hours of IL-4 stimulation. Data are represented as mean ± SD. **p ≤ 0.01; ***p ≤ 0.001; ****p ≤ 0.0001 by unpaired t test.

**Figure S4. Recombinant TREM2 antibody binds iMG in immunofluorescent assays.** (A) Control and IL-4 treated fixed iMG were immunostained using the recombinant TREM2 antibody or control IgG antibody and visualized using anti-human IgG fluorescent secondary antibody with DAPI counterstain. (B) Quantification of iMG immunostaining with IgG or TREM2 antibodies is graphed. Each data point represents the mean fluorescence of at least 300 iMG. Data are represented as mean ± SD. *p≤0.05; **p≤0.01; ***p≤0.01; ****p≤0.0001 analyzed by One-way ANOVA with post-hoc Tukey test. n=4/condition.

**Supplemental table 1.** Basal media components and differentiation factors

| **Compound** | **Vendor** | **Catalog #** |
| --- | --- | --- |
| DMEM/F12 + glutamax | Fisher Scientific | 10565-018 |
| Non-essential amino acid (NEAA) solution (100x) | Gibco/Fisher | 11140/11-140-050 |
| N2 (100x) | Gibco/Fisher | A1370701 |
| B27, serum free (50x) | Gibco/Fisher | 17504-044/17-504-044 |
| ITS-G (100x) | Fisher Scientific | 41400045 |
| Insulin | Sigma-Aldrich | I2643 |
| α-monothioglycerol | Sigma-Aldrich | M6145 |
| HEPES | Fisher Scientific | 15630-080 |
| IMDM | Fisher Scientific | 12440053 |
| HAM's F-12 nutrient mixture | Gibco/Fisher | 11765-054/11-765-054 |
| Poly(vinyl alcohol) | Sigma-Aldrich | P8136 |
| Lipid Concentrate (Chemically Defined) | MedChemExpress | HY-K3019 |
| ITS-X (100x) | Fisher Scientific | 51500-056 |
| l-Ascorbic acid 2-phosphate (AA2P) | Sigma-Aldrich | A8960 |
| GlutaMAX | Fisher Scientific | 3505-061 |
| β-Mercaptoethanol | ThermoFisher | 21985023 |
| Knock-out serum | ThermoFisher | A3181502 |
| Essential 8™ Medium | Gibco/Fisher | A1517001 |
| Y-27632 (Dihydrochloride) | StemCell | 72304 |
| Matrigel | Corning/Sigma | 356231 |
| Activin A | Peprotech | 120-14E |
| BMP-4 | Peprotech | 120-05 |
| CHiR | Sigma-Aldrich | SML1046 |
| VEGF _165_ | Peprotech | 100-20 |
| SB431542 | EMD Millipore | 616461 |
| bFGF | Peprotech | 100-18B |
| SCF | Peprotech | 300-07 |
| IL-6 | Peprotech | 200-06 |
| IL-3 | Peprotech | 200-03 |
| TPO | Peprotech | 300-18 |
| IL-34 | Peprotech | 200-34 |
| TGF-β1 | Peprotech | 100-21 |
| M-CSF | Peprotech | 300-25 |
| CX3CL1 | Peprotech | 300-31 |
| CD200 | Biolegend | 770006 |
| Citric Acid | Cell Signaling | 9871L |
| Tris HCl | Fisher Scientific | BP1757-100 |
| IL-4 | Peprotech | 200-04 |
| LPS | Sigma-Aldrich | L4391 |
| IFN-γ | Peprotech | 300-02 |
| pHrodo™ Red S. aureus BioParticles™ Conjugate | Invitrogen | A10010 |

**Supplemental table 2.** N2B27 media

| **N2B27 Media** | **For 250 mL** |
| --- | --- |
| DMEM/F-12 + GlutaMAX | 237.5 mL |
| N-2 Supplement (100X) | 2.5 mL |
| B-27™ Supplement (50X) | 5.0 mL |
| Non-essential amino acid (NEAA) solution (100x) | 2.5 mL |
| β-Mercaptoethanol | 454 μL |
| Basic FGF | 10 µg/mL |
| HEPES (1 M) | 3.75 mL |

**Supplemental table 3.** hES media

| **hES Media** | **For 50 mL** |
| --- | --- |
| DMEM/F-12 + GlutaMAX | 44.4 mL |
| Knock-out serum | 5 mL |
| Non-essential amino acid (NEAA) solution (100x) | 500 mL |
| β-Mercaptoethanol | 100 mL |

**Supplemental table 4.** iHPC basal media

| **Basal Media Name** | **Reagent** | **Volume (For 250 mL)** | **Final Concentration** |
| --- | --- | --- | --- |
| *iHPC* | IMDM | 119.35 mL |  |
|  | F‐12 | 119.35 mL |  |
|  | PVA (5%) | 50 μL | 10 mg/mL |
|  | Lipids (100x) | 250 uL | 0.1% |
|  | ITS-X (100x) | 5 mL | 2% |
|  | Monothioglycerol (150 mM) | 750 uL | 450 µM |
|  | AA2P (64 ng/mL) | 250 µL | 64 ng/µL |
|  | Glutamax (200 mM) | 2.5 mL | 2 mM |
|  | Non-essential amino acid (NEAA) solution (100x) | 2.5 mL | 1% |

**Supplemental table 5.** iMG basal media

| **Basal Media Name** | **Reagent** | **Volume** | **Final Concentration** |
| --- | --- | --- | --- |
| *iMG* | DMEM/F12 + glutamax (1x) | 234.5 mL |  |
|  | NEAA (100x) | 2.5 mL | 1x |
|  | N2 (100x) | 1.25 mL | 0.5x |
|  | B27, serum free (50x) | 2.5 mL | 0.5x |
|  | ITS-G (100x) | 5 mL | 2x |
|  | Insulin (5 µg/µL) | 250 µL | 5 µg/mL |
|  | Monothioglycerol (400 mM) | 250 µL | 400 µM |
|  | HEPES (1 M) | 3.75 mL | 15 mM |

**Supplemental table 6.** iHPC and iMG differentiation media and schedule

| **Microglia-like cell differentiation schedule** | | | |
| --- | --- | --- | --- |
| **Basal Media** | **Day** | **Factor** | **Final Concentration** |
| iHPC | 0 | BMP4 | 25 ng/mL |
|  |  | Activin A | 15 ng/mL |
|  |  | CHIR99021 | 1.5 µM |
|  | 2 | VEGF_165_ | 50 ng/mL |
|  |  | bFGF | 50 ng/mL |
|  |  | SB431542 | 10 µM |
|  |  | SCF | 50 ng/mL |
|  | 5 | VEGF_165_ | 50 ng/mL |
|  |  | bFGF | 50 ng/mL |
|  |  | SCF | 50 ng/mL |
|  |  | IL-3 | 10 ng/mL |
|  |  | IL-6 | 50 ng/mL |
|  |  | TPO | 50 ng/mL |
|  | 7 | VEGF_165_ | 50 ng/mL |
|  |  | bFGF | 50 ng/mL |
|  |  | SCF | 50 ng/mL |
|  |  | IL-3 | 10 ng/mL |
|  |  | IL-6 | 50 ng/mL |
|  |  | TPO | 50 ng/mL |
| iMG | 9-33  (every other day) | IL-34 | 100 ng/mL |
|  |  | TGFβ | 50 ng/mL |
|  |  | M-CSF | 25 ng/mL |
|  | 33+  (every other day) | IL-34 | 100 ng/mL |
|  |  | TGFβ | 50 ng/mL |
|  |  | M-CSF | 25 ng/mL |
|  |  | CX3CL1 | 100 ng/mL |
|  |  | CD200 | 100 ng/mL |

**Supplemental table 7.** Sequence of primers used in qRT-PCR analysis

| **Gene name** | **Forward primers (5' --> 3')** | **Reverse primers (5' --> 3')** |
| --- | --- | --- |
| ***IRF1*** | ATGCCCATCACTCGGATGC | CCCTGCTTTGTATCGGCCTG |
| ***IRF2*** | CATGCGGCTAGACATGGGTG | GCTTTCCTGTATGGATTGCCC |
| ***IRF3*** | AGAGGCTCGTGATGGTCAAG | AGGTCCACAGTATTCTCCAGG |
| ***IRF4*** | GCTGATCGACCAGATCGACAG | CGGTTGTAGTCCTGCTTGC |
| ***IRF5*** | GGGCTTCAATGGGTCAACG | GCCTTCGGTGTATTTCCCTG |
| ***IRF6*** | CCCCAGGCACCTATACAGC | TCCTTCCCACGGTACTGAAAC |
| ***IRF7*** | GCTGGACGTGACCATCATGTA | GGGCCGTATAGGAACGTGC |
| ***IRF8*** | ATGTGTGACCGGAATGGTGG | AGTCCTGGATACATGCTACTGTC |
| ***IRF9*** | GCCCTACAAGGTGTATCAGTTG | TGCTGTCGCTTTGATGGTACT |
| ***NANOG*** | TTTGTGGGCCTGAAGAAAACT | AGGGCTGTCCTGAATAAGCAG |
| ***POU5F1*** | CTGGGTTGATCCTCGGACCT | CCATCGGAGTTGCTCTCCA |
| ***PODXL*** | TCCCAGAATGCAACCCAGAC | GGTGAGTCACTGGATACACCAA |
| ***TMEM119*** | AGTCCTGTACGCCAAGGAAC | GCAGCAACAGAAGGATGAGG |
| ***P2RY12*** | AAGAGCACTCAAGACTTTAC | GGGTTTGAATGTATCCAGTAAG |
| ***TNFα*** | CCCAGGGACCTCTCTCTAATC | ATGGGCTACAGGCTTGTCACT |
| ***CXCL10*** | CCACGTGTTGAGATCATTGCT | TGCATCGATTTTGCTCCCCT |
| ***PLIN2*** | ATGGCATCCGTTGCAGTTGAT | GGACATGAGGTCATACGTGGAG |
| ***CD36*** | GGCTGTGACCGGAACTGTG | AGGTCTCCAACTGGCATTAGAA |
| ***MYLIP*** | GCAGGCGACTGGGAATCATAG | CGGTTTCTCAGGTTTAGCCAT |
| ***PPARG*** | GGGATCAGCTCCGTGGATCT | TGCACTTTGGTACTCTTGAAGTT |
| ***TNC*** | TCCCAGTGTTCGGTGGATCT | TTGATGCGATGTGTGAAGACA |
| ***LAMP3*** | GCGTCCCTGGCCGTAATTT | TGCTTGCTTAGCTGGTTGCT |
| ***CCL22*** | ATCGCCTACAGACTGCACTC | GACGGTAACGGACGTAATCAC |
| ***CCL1*** | CTCATTTGCGGAGCAAGAGAT | GCCTCTGAACCCATCCAACTG |
| ***NR4A3*** | TGCGTCCAAGCCCAATATAGC | GGTGTATTCCGAGCTGTATGTCT |
| ***LGI2*** | CTGAGCCTGGTAAATGGGACG | CCCGGATGATCGTGAATGAGTT |
| ***IL1-β*** | TTCGAGGCACAAGGCACAA | TGGCTGCTTCAGACACTTGAG |
| ***HEXB*** | CTCGCCCCGGAGAACTTCTA | GAAAGCATCACACTCTGACTGA |
| ***TYROBP*** | ACTGAGACCGAGTCGCCTTAT | ATACGGCCTCTGTGTGTTGAG |
| ***TREM2*** | TCTGAGAGCTTCGAGGATGC | GGGGATTTCTCCTTCAAGA |
|  |  |  |
